# Smoking and risk of negative outcomes among COVID-19 patients: A systematic review and meta-analysis

**DOI:** 10.18332/tid/132411

**Published:** 2021-02-04

**Authors:** Adinat Umnuaypornlert, Sukrit Kanchanasurakit, Don Eliseo III Lucero-Prisno, Surasak Saokaew

**Affiliations:** 1 School of Pharmaceutical Sciences, University of Phayao, Phayao, Thailand; 2 Unit of Excellence on Clinical Outcomes Research and IntegratioN, School of Pharmaceutical Sciences, University of Phayao, Phayao, Thailand; 3 Center of Health Outcomes Research and Therapeutic Safety, School of Pharmaceutical Sciences, University of Phayao, Phayao, Thailand; 4 Division of Pharmaceutical Care, Department of Pharmacy, Phrae Hospital, Phrae, Thailand; 5 Department of Global Health and Development, London School of Hygiene and Tropical Medicine, London, United Kingdom; 6 Unit of Excellence on Herbal Medicine, School of Pharmaceutical Sciences, University of Phayao, Phayao, Thailand; 7 Biofunctional Molecule Exploratory Research Group, Biomedicine Research Advancement Centre, School of Pharmacy, Monash University Malaysia, Bandar Sunway, Malaysia; 8 Novel Bacteria and Drug Discovery Research Group, Microbiome and Bioresource Research Strength, Jeffrey Cheah School of Medicine and Health Sciences, Monash University Malaysia, Bandar Sunway, Malaysia

**Keywords:** smoking, disease severity, death, COVID-19, coronavirus

## Abstract

**INTRODUCTION:**

COVID-19 has major effects on the clinical, humanistic and economic outcomes among patients, producing severe symptoms and death. Smoking has been reported as one of the factors that increases severity and mortality rate among COVID-19 patients. However, the effect of smoking on such medical outcomes is still controversial. This study conducted a comprehensive systematic review and meta-analysis (SR/MA) on the association between smoking and negative outcomes among COVID-19 patients.

**METHODS:**

Electronic databases, including PubMed, EMBASE, Cochrane Library, Science Direct, Google Scholar, were systematically searched from the initiation of the database until 12 December 2020. All relevant studies about smoking and COVID-19 were screened using a set of inclusion and exclusion criteria. The Newcastle–Ottawa Scale was used to assess the methodological quality of eligible articles. Random meta-analyses were conducted to estimate odds ratios (ORs) with 95% confidence interval (CIs). Publication bias was assessed using the funnel plot, Begg’s test and Egger’s test.

**RESULTS:**

A total of 1248 studies were retrieved and reviewed. A total of 40 studies were finally included for meta-analysis. Both current smoking and former smoking significantly increase the risk of disease severity (OR=1.58; 95% CI: 1.16–2.15, p=0.004; and OR=2.48; 95% CI: 1.64–3.77, p<0.001; respectively) with moderate appearance of heterogeneity. Similarly, current smoking and former smoking also significantly increase the risk of death (OR=1.35; 95% CI: 1.12–1.62, p=0.002; and OR=2.58; 95% CI: 2.15–3.09, p<0.001; respectively) with moderate appearance of heterogeneity. There was no evidence of publication bias, which was tested by the funnel plot, Begg’s test and Egger’s test.

**CONCLUSIONS:**

Smoking, even current smoking or former smoking, significantly increases the risk of COVID-19 severity and death. Further causational studies on this association and ascertianing the underlying mechanisms of this relation is warranted.

## INTRODUCTION

Since December 2019, there has been an outbreak of pneumonia of unknown etiology that was first reported in Wuhan, Hubei Province, China. Following the outbreak, a novel coronavirus SARS-CoV-2 disease, COVID-19, was identified by the World Health Organization (WHO) as the causative virus for the pandemic in China and other parts of the world with more than 30 million cases of infection and 0.9 million deaths globally^1^. In addition, COVID-19 pandemic caused poor mental health and quality of life, as reported. This pandemic is seen to be far from over and there is a continuing resurgence in many countries. The COVID-19 pandemic has caused panic and anxiety because of the increasing number of COVID-19 cases worldwide^2,3^. Furthermore, COVID-19 has had a significant global economic impact and a huge burden on healthcare resources^4^.

Smoking has been assumed to be associated with adverse disease prognosis, as extensive evidence has highlighted the negative impact of tobacco use on lung health. It is also found to be detrimental to the immune system and its responsiveness to infections, making smokers more vulnerable to infectious diseases^5^. Smoking increases the risk and severity of pulmonary infections because of damage to upper airways and a decrease in pulmonary immune function^6^. It still remains controversial, however, if smoking results in severe symptoms and death among COVID-19 patients. Some previous studies reported a significant association between current smoking, former versus never smoking with COVID-19 negative outcomes^7–10^. The differences between risk of severity and death between former and never smoker COVID-19 patients have not been shown^11–13^. Because of small sample sizes included in these previous studies and differing definitions of disease severity, existing systematic reviews and meta-analyses found limited evidence suggesting that the risk of COVID-19 infection maybe lower among smokers compared to non-smokers, albeit from highly heterogeneous studies^14–18^.

There were a number of factors related to the severity of COVID-19 and the mortality rate, including: older age (>65 years), comorbidities (e.g. hypertension, diabetes), organ dysfunction, lymphopenia, high cytokines, and weak immune responses^19–22^. Especially, older age was associated with a dramatically higher risk of severe COVID-19. For example, the case fatality rate in three databases exceeded 1% around the age of 50–55 years, but was 10% above 80–85 years (≥70 years in Italy)^23^. Males aged >65 years, and smoking patients, face greater risk of developing a severe or critical condition^19^. A previous meta-analysis showed that all age groups had significantly higher mortality compared to their immediately younger age group, with the largest increase in mortality risk observed in patients with ages 60–69 compared to 50–59 years^24^. This fact could be influenced by both the aging process and the high prevalence in frailty and comorbidities among the older people, which contribute to a decrease in their functional capacity.

Given the unclear evidence about smoking in COVID-19 infected patients aged ≤65 years, we conducted a comprehensive SR/MA to determine the association between smoking and disease severity in COVID-19 infected patients by including all eligible studies. Systematic searching of databases for available evidence and careful definition of disease severity was performed for a rigorous summary of the conclusions.

## METHODS

### Protocol and registration

The systematic review and meta-analysis were performed in line with the Preferred Reporting Items for Systematic Reviews and Meta-analyses (PRISMA) statement^25^. This research was registered with PROSPERO (Registration Number CRD42020186638). Patients and the public were not involved in this study. The systematic review and meta-analysis are exempt from ethics approval because data were collected and synthesized from previous studies. The patient data are anonymized and available in the public domain. The authors followed applicable EQUATOR Network (https://www.equator-network.org) guidelines during the conduct of research project.

### Data sources and search strategy

To find relevant studies, scientific databases including Embase, PubMed, Science Direct, Google Scholar and Cochrane Library databases were systematically searched from their inception to 12 December 2020. Medical Subject Headings (MeSH) were used whenever applicable. Bibliographic lists of related articles were explored. The search strategy was carried out with the following keywords: [tobacco OR smok*] AND [covid OR coronavirus OR sars cov*] with slight adjustments depending on the database. There was no study design and language restriction. Additionally, extra searches were performed in the reference lists of included studies to avoid missing any article (Supplementary file Table S1).

### Study selection

All relevant articles that reported clinical characteristics and epidemiological information on smoking among COVID-19 infected patients were included in the analysis. All articles with any design (randomized controlled trials and observational studies) were included. Animal studies, reviews, commentaries, editorials, expert opinions, letters, conference meeting abstracts, case reports, case series, systematic reviews and meta-analyses were excluded. Studies with the same participants that did not include effect estimates or had insufficient data to measure effect estimates were also eliminated. Articles with explicit involvement with the tobacco industry were excluded.

### Outcomes measures

The primary outcome was disease severity among COVID-19 patients with a history of smoking. The secondary outcome was death among COVID-19 patients with a history of smoking. The term ‘disease severity’ includes clinical presentations based on physical examinations and laboratory results, and other medical records, as diagnosed and described by physicians.

Disease severity was defined by any of the following criteria.

1. Patients who required ICU care^26^.
2. Severe case as defined by the American Thoracic Society guidelines for community-acquired pneumonia^22^.
3. Severe stage, if any of the following criteria existed:
  a) shortness of breath, respiratory rate ≥30 times/min; b) oxygen saturation <93% in resting state; c) PaO2/FiO2 ≤300 mmHg. CT imaging showed significant lesion progression >50% within 24 to 48 h; d) respiratory failure requiring mechanical ventilation; e) shock; and f) complications with other organ failure requiring ICU care^27^.
4. Severe cases were patients needed supplemental oxygen therapy^28^.
5. Severe cases or patients with Acute Respiratory Distress Syndrome (ARDS) having PaO2/FiO2 ≤300 mmHg^29^.
6. Severe or critical patients as defined by the General Office of National Health Commission of China, version 5 (2020)^30^.

In cases where smoking status did not specify type of smoking, it was taken to be current smoking.

### Data extraction and quality assessment

Two investigators (AU and SK) independently screened each title, abstract and full-text article for potentially eligible studies. Discrepancies were resolved by discussions with a third investigator (SS). All extracted data were independently reviewed by two investigators (AU and SK). The following information was extracted from each study: setting, region, design, sample size, demographic characteristics of participants (age, sex), details of intervention/exposure (smoking status: current or former smoker), and details of outcomes (disease severity: severe or critical vs non-severe; death), and number of COVID-19 patients. The quality of individual studies was appraised independently using the Newcastle–Ottawa Scale (NOS)^31^. The NOS assigns a maximum of 9 points, with studies having a total score of ≥7 defined as high quality.

### Statistical analysis

We computed odds ratio (OR) and 95% confidence interval (CI) for each study using the number of smokers (former or current) and never smoker with pre-specified outcomes (severity and death). The pool effects were combined using random-effect model. Heterogeneity was investigated using Cochran’s Q statistic and I^2^. Cochran’s Q statistic with an alpha value of 0.10 was chosen to designate heterogeneity amongst trials for each analysis. Heterogeneity level was assigned as: I^2^ >75%, 25–75%, and <25% to indicate high, moderate, and low level, respectively^31^. In the case where heterogeneity existed, an attempt to explore possible sources of heterogeneity was made. Publication bias was assessed using Begg’s test, Egger’s test, and funnel plot^32–34^. A p<0.05 in publication bias tests was suggestive of publication bias. When publication bias was found, the trim-and-fill method was used^35^.

### Sensitivity and subgroup analysis

To appraise the robustness of our analysis, the sensitivity analysis for unmeasured confounding was used. Subgroup analyses were conducted by age differences between groups, current and former exposure to smoking, and quality of the studies. Meta-regression analysis was performed using random-effects meta-regression, *metareg* command in STATA software^36^, adjusting for study characteristics (covariates) on pooled outcome. The following potential moderator variables: age (>65 years), hypertension and diabetes mellitus were included for meta-regression analysis.

## RESULTS

### Search results and characteristics of studies included

**Figure 1:**
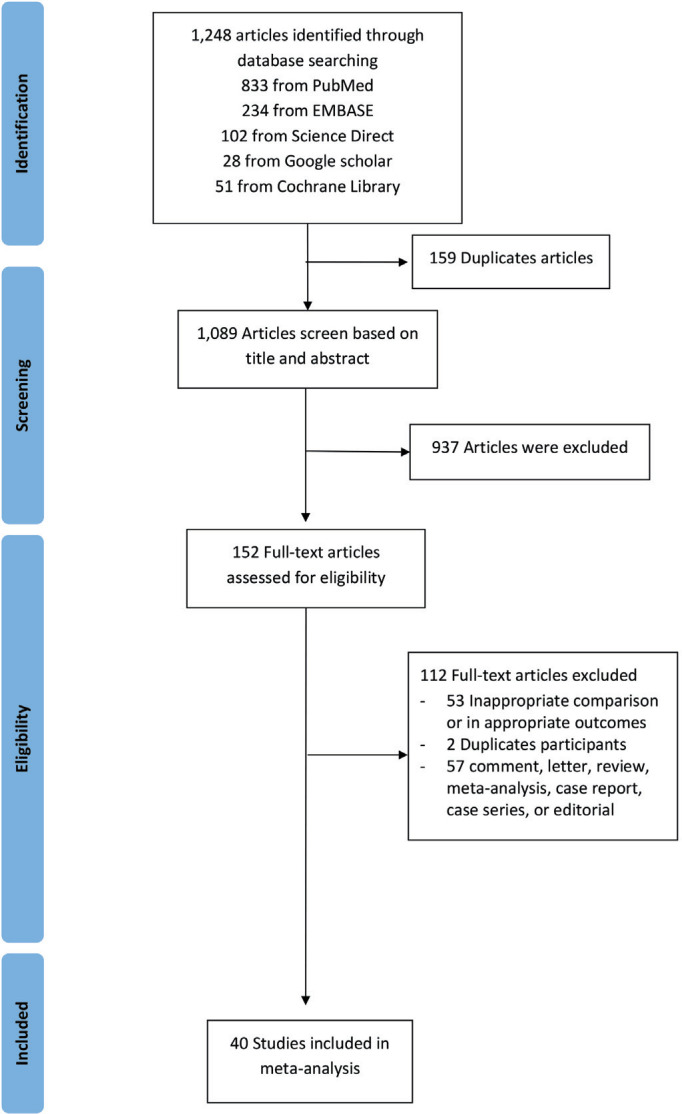
PRISMA flow diagram

In the initial search, 1248 articles were retrieved from all databases. Of these, 159 were eliminated that were found to be duplicates. All articles were screened using the title and abstract. After evaluating the abstracts, 937 studies were excluded due to their data being irrelevant to our objective. After evaluating the full text, a total of 40 studies with 369287 COVID-19 infected patients were included in the meta-analysis (Figure 1). The important characteristics and outcomes of the included articles were collated (Table 1). Of 40 articles, 19 were conducted in China^21,22,27,29,30,37–50^, one in Kuwait^26^, one in Korea^28^, one in Mexico^51^, one in Japan^52^, two in Spain^53,54^, three in Italy^55–57^, and twelve in the USA^33,58–68^. Most articles were retrospective studies. The mean age of the patients in the included studies was 54.10 years. Nineteen studies defined outcomes as disease severity^22,27–30,37–39,42,43,46–50,58,60,62,63^. Seventeen studies defined outcomes as death^21,33,40,44,45,51–57,59,65–68^. Four studies used both disease severity and death^26,41,61,64^. All studies defined smoking status as current smoker. Eleven studies included former smokers and current smokers^22,33,43,44,47,57,58,61–63,67^.

### Quality assessment

Newcastle–Ottawa scale was used to assess the methodological quality of the 40 studies. Results showed 12 studies receiving ≥7 stars^26,44,49,55–57,59–62,64,68^, and the remaining studies receiving <7 stars (Supplementary Table S2).

### Synthesis of results

**Figure 2:**
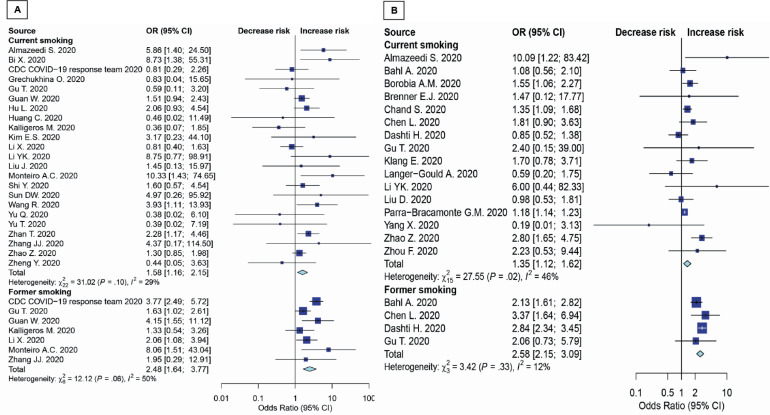
Forest plots showing odds ratio of disease severity (A) and death (B) among younger smokers (≤65 years)

The results in younger patients (≤65 years) showed that both current smoking and former smoking significantly increase the risk of disease severity (OR=1.58; 95% CI: 1.16–2.15, p=0.004; and OR=2.48; 95% CI: 1.64–3.77, p<0.001; respectively) (Figure 2A). Moreover, both current smoking and former smoking also significantly increase the mortality risk in COVID-19 patients (OR=1.35; 95% CI: 1.12–1.62, p=0.002; and OR=2.58; 95% CI: 2.15–3.09; p<0.001; respectively) with moderate appearance of heterogeneity (Figure 2B).

### Sensitivity and subgroup analyses

**Figure 3:**
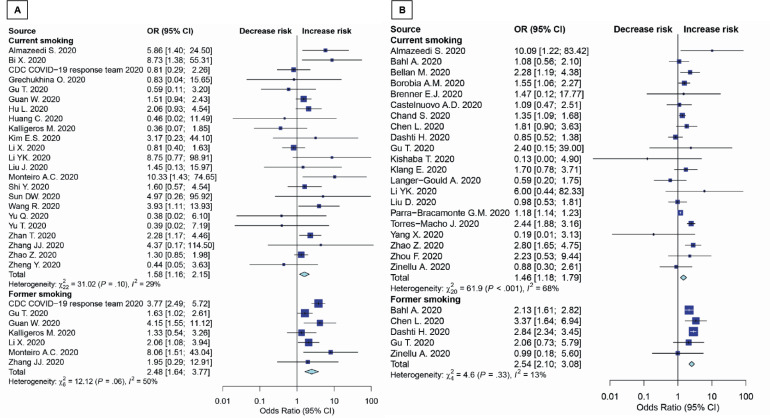
Forest plot showing odds ratio of disease severity (A) and death (B) among all age smokers

These analyses were conducted for patients >65 years. Results showed that both current smoking and former smoking significantly increase the risk of death (OR=1.46; 95% CI: 1.18–1.79, p=0.002; and OR=2.54; 95% CI: 2.10–3.08, p<0.001; respectively) (Figure 3B). There were no studies with patients aged >65 years in severity outcome (Figure 3A). The sensitivity analysis for unmeasured confounding for death outcome remained substantial (OR=1.38; 95% CI: 1.12–1.71, p=0.003). The subgroup results were consistent with the main study results mentioned above. Details are shown in Table 2. Subgroup analyses were conducted using average age groups (≤65, >65 years), age differences between groups, current and former exposure to smoking, and the quality of the studies. Both average age groups had higher death rate than never smoker. For the disease severity among current smokers, the OR for the random-effects model in the different age groups was 1.97 (95% CI: 1.21–3.22, p=0.007) and 1.41 (95% CI: 1.01–1.97, p=0.046) in similar age groups. For the disease severity among former smokers, the OR for the random-effects model in the different age groups was 1.77 ( 95% CI: 1.22–2.58, p=0.003) and 3.05 (95% CI: 1.11–8.37, p=0.030) in similar age groups. For death among current smokers, the OR for the random-effects model in the different age groups was 1.53 (95% CI: 1.23–1.90, p<0.001). For the death among former smokers, the OR for the randomeffects model in the different age groups was 2.54 (95% CI: 2.10–3.08, p<0.001). While the death OR from the random-effects model in the stars ≥7 group (NOS quality of study) was 1.86 (95% CI: 1.35–2.55, p<0.001) and 1.52 (95% CI: 1.14–2.02, p=0.004) for stars <7 group. The severity OR from the randomeffects model in the stars <7 group was 2.17 (95% CI: 1.57–3.00, p<0.001) (Table 2).

**Table 1:** General characteristics of 40 studies included

*Author and Year*	*Location*	*Study design*	*Baseline participant characteristics*		*Type of smoker ^a^*	*Outcomes measures*	*OR (95% CI)*	*Quality of studies ^b^*
			*Participants*	*Age (years) Median or Mean (SD)*				
Almazeedi S. (2020)	Kuwait	Retrospective cohort study	1096	41	Current	Disease severity Death	5.86 (1.40–24.47) 10.09 (1.22–83.40)	7/9
Bahl A. (2020)	USA	Multicenter cohort study	1461	62	Current Former	Death	1.08 (0.54–2.04) 2.13 (1.61–2.82)	6/9
Bellan M. (2020)	Italy	Retrospective study	312	71	Current	Death	2.28 (1.18–4.35)	7/9
Bi X. (2020)	China	Retrospective study	113	46	Current	Disease severity	8.73 (1.49–59.80)	6/9
Borobia A. M. (2020)	Spain	Retrospective study	2226	61	Current	Death	1.55 (1.05–2.25)	6/9
Brenner E.J. (2020)	USA and other countries	Retrospective study	525	41	Current	Death	1.47 (0.12–17.53)	6/9
Castelnuovo A.D. (2020)	Italy	Retrospective observational study	1842	67 (12.96)	Current	Death	1.09 (0.47–2.49)	7/9
CDC response team (2020)	USA	Retrospective study	6637	≥19	Current Former	Disease severity	0.81 (0.26–1.99) 3.77 (2.46–5.65)	5/9
Chand S. (2020)	USA	Retrospective study	300	58.2 (12.6)	Current	Death	1.35 (1.09–1.68)	6/9
Chen L. (2020)	China	Retrospective study	1859	59	Current Former	Death	1.81 (0.87–3.50) 3.37 (1.59–6.74)	8/9
Dashti H. (2020)	USA	Retrospective study	12347	48	Current Former	Death	0.85 (0.51–1.34) 2.84 (2.34–3.46)	6/9
Grechukhina O. (2020)	USA	Retrospective cohort study	141	30	Current	Disease severity	0.83 (0.02–7.11)	7/9
Gu T. (2020)	USA	Retrospective cohort study	766	47	Current Current Former Former	Disease severity Death Disease severity Death	0.59 (0.11–3.23) 2.40 (0.15–39.60) 1.63 (1.02–2.61) 2.06 (0.73–5.77)	8/9
Guan W. (2020)	China	Retrospective study	1085	47	Current Former	Disease severity	1.51 (0.93–2.40) 4.15 (1.51–10.90)	6/9
Hu L. (2020)	China	Retrospective study	323	61	Current	Disease severity	2.06 (0.96–4.66)	6/9
Huang C. (2020)	China	Retrospective study	41	49	Current	Disease severity	0.46 (0.01–5.40)	6/9
Kalligeros M. (2020)	USA	Retrospective study	103	60	Current Former	Disease severity	0.36 (0.06–1.59) 1.33 (0.54–3.24)	8/9
Kim E.S. (2020)	Korea	Retrospective study	28	42.6 (13.4)	Current	Disease severity	3.17 (0.19–37.39)	5/9
Kishaba T. (2020)	Japan	Single-center retrospective cohort study	7	74	Current	Death	0.13 (0.00–3.08)	6/9
Klang E. (2020)	USA	Retrospective study	572	46.5	Current	Death	1.70 (0.80–3.80)	8/9
Langer-Gould A. (2020)	USA	Retrospective cohort study	93	59.3	Current	Death	0.59 (0.20–1.68)	7/9
Li X. (2020)	China	Ambispective cohort study	548	60	Current Former	Disease severity	0.81 (0.4–1.61) 2.06 (1.09–3.99)	6/9
Li YK. (2020)	China	Retrospective study	25	51	Current	Disease severity Death	8.75 (0.89–113.30) 6.00 (0.47–87.66)	6/9
Liu D. (2020)	China	Retrospective study	599	63	Current	Death	0.98 (0.52–1.78)	6/9
Liu J. (2020)	China	Retrospective study	40	48.7	Current	Disease severity	1.45 (0.12–14.56)	6/9
Monteiro A.C. (2020)	USA	Retrospective observational cohort study	112	61	Current Former	Disease severity	10.33 (1.43–74.67 8.06 (1.51–43.06)	6/9
Parra-Bracamonte G. M. (2020)	Mexico	Retrospective study	331298	44	Current	Death	1.18 (1.13–1.22)	6/9
Shi Y. (2020)	China	Retrospective study	487	46	Current	Disease severity	1.60 (0.52–4.17)	6/9
Sun DW. (2020)	China	Retrospective study	57	64	Current	Disease severity	4.97 (0.61–227.20)	6/9
Torres-Macho J. (2020)	Spain	Retrospective observational study	1968	67	Current	Death	2.44 (1.89–3.17)	6/9
Wang R. (2020)	China	Retrospective study	125	42	Current	Disease severity	3.93 (1.08–13.56)	6/9
Yang X. (2020)	China	Retrospective observational study	52	51.9	Current	Death	0.19 (0.01–2.66)	6/9
Yu Q. (2020)	China	Multicenter cohort study	421	48	Current	Disease severity	0.38 (0.01–2.58)	7/9
Yu T. (2020)	China	Cross-sectional multicenter clinical study	95	40 (15.88)	Current	Disease severity	0.39 (0.01–3.40)	6/9
Zhan T. (2020)	China	Retrospective study	405	56	Current	Disease severity	2.28 (1.17–4.47)	6/9
Zhang JJ. (2020)	China	Retrospective study	140	57	Current Former	Disease severity	4.37 (0.34–232.00) 1.95 (0.31–13.78)	6/9
Zhao Z. (2020)	USA	Retrospective study	641	60	Current	Death Disease severity	2.80 (1.64–4.72) 1.30 (0.85–1.97)	7/9
Zheng Y. (2020)	China	Retrospective study	73	43	Current	Disease severity	0.44 (0.04–2.73)	6/9
Zhou F. (2020)	China	Retrospective cohort study	191	56	Current	Death	2.23 (0.51–9.17)	6/9
Zinellu A. (2020)	Italy	Retrospective study	94	72	Current Former	Death	0.88 (0.29–2.55) 0.99 (0.15–4.80)	7/9

SD: standard deviation. CI: confidence interval.

a Type of smoker: current smoker defined as adult who has smoked 100 cigarettes in lifetime and who currently smokes cigarettes; former smoker defined as adult who has smoked at least 100 cigarettes in his/her lifetime but who had quit smoking at the time of interview.

b Assessed by Newcastle-Ottawa Scale.

**Table 2:** Sensitivity and subgroup analyses

*Characteristics*	*All negative outcomes*			*Disease severity*			*Death*		
	*OR (95% CI)*	*Heterogeneity*		*OR (95% CI)*	*Heterogeneity*		*OR (95% CI)*	*Heterogeneity*	
		*I^2^ (%)*	*p*		*I^2^ (%)*	*p*		*I^2^ (%)*	*p*
**Models**									
Fixed effects model	1.28 (1. 24–1.33)	75.8	<0.001	1.87 (1.58–2.20)	44.5	0.005	1.26 (1.22–1.31)	83.8	<0.001
Random effects model	1.73 (1.45–2.05)	75.8	<0.001	1.87 (1.43–2.44)	44.5	0.005	1.63 (1.30–2.04)	83.8	<0.001
**Age** (years)									
Overall	1.73 (1.45–2.05)	75.8	<0.001	1.87 (1.43–2.44)	44.5	0.005	1.63 (1.30–2.04)	83.8	<0.001
≤65	1.74 (1.45–2.44)	75.3	<0.001	1.87 (1.43–2.44)	44.5	0.005	1.64 (1.28–2.10)	84.6	<0.001
>65	1.65 (1.04–2.62)	45.5	0.103	N/A	N/A	N/A	1.65 (1.04–2.62)	45.5	0.103
**Age different between groups of current smokers**									
Different	1.61 (1.32–1.96)	65.0	<0.001	1.97 (1.21–3.22)	40.3	0.072	1.53 (1.23–1.90)	71.0	<0.001
Similar	1.25 (0.84–1.88)	18.6	0.266	1.41 (1.01–1.97)	5.4	0.390	0.52 (0.18–1.48)	0.0	<0.001
**Age different between groups of former smokers**									
Different	2.36 (1.97–2.83)	15.4	0.309	1.77 (1.22–2.58)	0.0	0.844	2.54 (2.10–3.08)	13.1	0.330
Similar	3.05 (1.11–8.37)	58.0	0.093	3.05 (1.11–8.37)	58.0	0.093	N/A	N/A	N/A
**Quality of the study** (NOS)									
stars ≥7	1.65 (1.28–2.12)	32.5	0.081	1.35 (0.93–1.98)	22.1	0.253	1.86 (1.35–2.55)	30.8	0.145
stars <7	1.79 (1.4–2.23)	81.7	<0.001	2.17 (1.57–3.00)	42.6	0.019	1.52 (1.14–2.02)	89.7	<0.001
**Omitted unadjusted OR studies**									
Random effects model	1.38 (1.12–1.71)	0.8	0.402	N/A	N/A	N/A	1.38 (1.12–1.71)	0.8	0.402

OR: odds ratio. CI: confidence interval. N/A: not available. NOS: Newcastle-Ottawa Scale.

Meta-regression was performed to investigate the following potential moderator variables: age (>65 years), hypertension and diabetes mellitus. No significant moderators of primary and secondary outcomes with studies contributing data emerged, including age >65 years, hypertension, and diabetes mellitus (Supplementary file Table S5).

### Publication bias of included studies

An appraisal of publication bias was conducted. There was no apparent publication bias as determined by the symmetric funnel plot, and Begg’s and Egger’s tests revealed no significant difference in all age groups and all outcomes (Supplementary file Figures S1–S6).

## DISCUSSION

### Summary of evidence

Both current and former smoking significantly increase the risk of disease severity (OR=1.58; 95% CI: 1.16–2.15, p=0.004; and OR=2.48; 95% CI: 1.64–3.77, p<0.001; respectively). Moreover, both current and former smoking also significantly increase the mortality risk among ≤65 years COVID-19 patients (OR=1.35; 95% CI: 1.12–1.62, p=0.002; and OR=2.58; 95% CI: 2.15–3.09, p<0.001; respectively).

We performed a comprehensive SR/MA to assess the possible association between disease severity and death among smokers with COVID-19. According to our analysis, with the biggest sample size, smoking is a risk factor for disease severity and death in COVID-19 patients. Current smokers have 1.58 times the odds of disease severity than never smokers. Remarkably, former smokers have 2.48 times odds of disease severity than never smokers. For death outcome, current and former smoking also significantly increase the risk of death by 1.35 and 2.58 times, respectively.

The most likely mechanism for the potential increase in the risk might be associated with the angiotensin II conversion enzyme-2 (ACE2) receptor, which is in the mucosal epithelial cell and lung alveolar tissue and found to be related to infections with COVID-19. The infection by the host virus attaching to the ACE2 receptors is probably a key step for coronavirus infection. The ACE2 gene expression is heightened in both current and former smokers compared to never smokers in a sample of patients with lung adenocarcinoma, after adjusting for age, gender, and ethnicity^5,6,69^. This might be a reason why former smokers have higher odds of negative outcomes than never smokers. On the contrary, the findings indicated that current smoking was less likely to have negative outcomes compared with former smoking. These might be due to the following reasons. First, the under-reporting of the current smoking status. Most studies reported smoking history instead of current smoking, which might include former smokers and therefore underestimate current smoking status among COVID-19 patients^70^. Second, former smokers have longer exposure period or accompanying diseases such as asthma, COPD due to smoking^18^. As a result, former smoking showed higher risk of negative outcomes compared with current smoking.

Although a previous systematic review examined the association between smoking and overall negative outcomes among COVID-19 patients, it was limited to only Chinese patients^12^. Another systematic review did not summarize the results as a meta-analysis^13^. One study demonstrated only the prevalence of smokers among patients hospitalized with COVID-19^71^ while in another study, the authors retrieved the studies from only one database and the definition of smoking was unclear^8^. One focused on chronic obstructive pulmonary disease (COPD) and ongoing smoking history^17^. One meta-analysis included just four selected studies of fair quality, which found that current smokers were more likely to develop severe COVID-19 illness compared to never smokers. But no significant difference was observed between former and never smokers. They also conducted a meta-analysis using two studies deemed to be of fair quality. So they found no significant difference between the risk of death from COVID-19 either between current and never smokers, or former and never smokers^11^. Finally, all literature collected did not exclude people aged >65 years, which could be a disruptive variable to the study results.

The research question requires well-designed population-based studies that control for age and relevant underlying risk factors. To our best knowledge, this study is the first comprehensive meta-analysis to assess the potential association between former and current smokers and negative outcomes of COVID-19, with the biggest sample size.

### Strengths and limitations

This study has several strengths. First, we performed a comprehensive search of major databases (Embase, PubMed, Science Direct, Google Scholar and Cochrane), which is a standard method for conducting a systematic review. Second, we employed a comprehensive search strategy with no restrictions on language and study design. Third, this meta-analysis adheres to the standard methodology of systematic reviews and meta-analyses as required by the PRISMA checklist. Fourth, our study covered updated evidence and was conducted using the appropriate statistical methods for analysis. Finally, the robustness including sensitivity-analysis, subgroup-analysis and meta-regression illustrated that the results remain unchanged.

The study also has some limitations. First, all studies included were observational studies which might have residual confounders; however, this kind of study design reflects a real-world situation for evaluating the association between smoking and disease severity or death in COVID-19 patients. We also used adjusted data from the included studies as much as possible. Nevertheless, there were only non-adjusted data available in some studies. Thus, the residual confounders might distort associations and conclusions. For example, obesity, diabetes, hypertension, asthma and age were reported to increase the risk of severity of COVID-19^72–74^. We, therefore, analyzed using meta-regression and found that the conclusion remained the same. Second, we searched five major databases, which might not have covered all relevant studies. Nonetheless, after applying Begg’s test, Egger’s test, and a funnel plot, we found no evidence of publication bias. Third, the definitions of severity in each study were slightly different and this is a broad exploratory meta-analysis, which might distort the association between smoking and outcome in COVID-19 patients. Therefore, the results should be interpreted cautiously. However, from another perspective, the effects of smoking in our analysis were consistent across studies, which may indicate high generalizability of the results to any circumstances. Fourth, even key important factors that may potentially affect our findings were number of cigarettes smoked, nicotine addiction level, and the length of time after quitting until COVID-19 infection, which were not reported in the included studies. Nevertheless, our comprehensive sensitivity analysis showed a negative association of smoking on the outcomes.

### Further research directions

Well-designed longitudinal population-based studies are needed to address questions about the risk of infection by SARS-CoV-2 and the risk of hospitalization with COVID-19. Stronger evidence coming from smoking status data that are systemically recorded and analyzed among COVID-19 patients are needed. Some factors such as number of cigarettes smoked, nicotine addiction level, and the length of time after quitting until COVID-19 infection should be collected.

## CONCLUSIONS

Smoking is confirmed to be a risk factor for the negative progression of COVID-19, particularly on disease severity and death. Both current and former smokers have higher odds of disease severity than never smokers. Given the well-established harm associated with tobacco use, smoking cessation is recommended for all smokers and avoidance of secondhand smoke by non-smokers.

## References

[1] World Health Organization Weekly operational update on COVID-19 - 18 September.

[2] Qiu J, Shen B, Zhao M, Wang Z, Xie B, Xu Y (2020). A nationwide survey of psychological distress among Chinese people in the COVID-19 epidemic: Implications and policy recommendations. Gen Psychiatr.

[3] Zhang Y, Ma ZF (2020). Impact of the COVID-19 pandemic on mental health and quality of life among local residents in Liaoning Province, China: A cross-sectional study. Int J Environ Res Public Health.

[4] Asian Development Bank An Updated Assessment of the Economic Impact of COVID-19.

[5] Strzelak A, Ratajczak A, Adamiec A, Feleszko W (2018). Tobacco smoke induces and alters immune responses in the lung triggering inflammation, allergy, asthma and other lung diseases: A mechanistic review. Int J Environ Res Public Health.

[6] Arcavi L, Benowitz NL (2004). Cigarette smoking and infection. Arch Intern Med.

[7] Alqahtani JS, Oyelade T, Aldhahir AM (2020). Prevalence, Severity and Mortality associated with COPD and Smoking in patients with COVID-19: A Rapid Systematic Review and Meta-Analysis. PLoS One.

[8] Patanavanich R, Glantz SA Smoking is associated with worse outcomes of COVID-19 particularly among younger adults: A systematic review and meta-analysis. medRxiv.

[9] Kozak R, Prost K, Yip L, Williams V, Leis JA, Mubareka S (2020). Severity of coronavirus respiratory tract infections in adults admitted to acute care in Toronto, Ontario. J Clin Virol.

[10] Guo FR (2020). Smoking links to the severity of COVID-19: An update of a meta-analysis. J Med Virol.

[11] Simons D, Shahab L, Brown J, Perski O The association of smoking status with SARS-CoV-2 infection, hospitalisation and mortality from COVID-19: A living rapid evidence review (version 4). Qeios.

[12] Lippi G, Henry BM (2020). Active smoking is not associated with severity of coronavirus disease 2019 (COVID-19). Eur J Intern Med..

[13] Vardavas CI, Nikitara K (2020). COVID-19 and smoking: A systematic review of the evidence. Tob Induc Dis.

[14] Patanavanich R, Glantz SA (2020). The Theoretical Problems Do Not Materially Affect the Results of Our Meta-analysis of Smoking and Covid-19 Disease Progression. Nicotine Tob Res.

[15] Grundy EJ, Suddek T, Filippidis FT, Majeed A, Coronini-Cronberg S (2020). Smoking, SARS-CoV-2 and COVID-19: A review of reviews considering implications for public health policy and practice. Tob Induc Dis.

[16] Reddy RK, Charles WN, Sklavounos A, Dutt A, Seed PT, Khajuria A (2020). The effect of smoking on COVID-19 severity: A systematic review and meta-analysis. J Med Virol.

[17] Zhao Q, Meng M, Kumar R (2020). The impact of COPD and smoking history on the severity of COVID-19: A systemic review and meta-analysis. J Med Virol.

[18] Gülsen A, Yigitbas BA, Uslu B, Drömann D, Kilinc O (2020). The Effect of Smoking on COVID-19 Symptom Severity: Systematic Review and Meta-Analysis. Pulm Med.

[19] Zheng Z, Peng F, Xu B (2020). Risk factors of critical & mortal COVID-19 cases: A systematic literature review and meta-analysis. J Infect.

[20] Williamson EJ, Walker AJ, Bhaskaran K (2020). Factors associated with COVID-19-related death using OpenSAFELY. Nature.

[21] Yang X, Yu Y, Xu J (2020). Clinical course and outcomes of critically ill patients with SARS-CoV-2 pneumonia in Wuhan, China: a single-centered, retrospective, observational study. Lancet Respir Med.

[22] Li X, Xu S, Yu M (2020). Risk factors for severity and mortality in adult COVID-19 inpatients in Wuhan. J Allergy Clin Immunol.

[23] Onder G, Rezza G, Brusaferro S (2020). Case-Fatality Rate and Characteristics of Patients Dying in Relation to COVID-19 in Italy. JAMA.

[24] Bonanad C, García-Blas S, Tarazona-Santabalbina F (2020). The Effect of Age on Mortality in Patients With COVID-19: A Meta-Analysis With 611,583 Subjects. J Am Med Dir Assoc.

[25] Liberati A, Altman DG, Tetzlaff J (2009). The PRISMA statement for reporting systematic reviews and meta-analyses of studies that evaluate healthcare interventions: explanation and elaboration. BMJ.

[26] Almazeedi S, Al-Youha S, Jamal MH (2020). Characteristics, risk factors and outcomes among the first consecutive 1096 patients diagnosed with COVID-19 in Kuwait. EClinicalMedicine.

[27] Bi X, Su Z, Yan H (2020). Prediction of severe illness due to COVID-19 based on an analysis of initial Fibrinogen to Albumin Ratio and Platelet count. Platelets.

[28] Kim ES, Chin BS, Kang CK (2020). Clinical course and outcomes of patients with severe acute respiratory syndrome coronavirus 2 infection: A preliminary report of the first 28 patients from the Korean Cohort Study on COVID-19. J Korean Med Sci.

[29] Yu T, Cai S, Zheng Z (2020). Association Between Clinical Manifestations and Prognosis in Patients with COVID-19. Clin Ther.

[30] Hu L, Chen S, Fu Y (2020). Risk Factors Associated With Clinical Outcomes in 323 Coronavirus Disease 2019 (COVID-19) Hospitalized Patients in Wuhan, China. Clin Infect Dis.

[31] Higgins JPT, Thompson SG, Deeks JJ, Altman DG (2003). Measuring inconsistency in meta-analyses. BMJ.

[32] Begg CB, Berlin JA (1989). Publication bias and dissemination of clinical research. J Natl Cancer Inst.

[33] Bahl A, Van Baalen MN, Ortiz L (2020). Early predictors of in-hospital mortality in patients with COVID-19 in a large American cohort. Intern Emerg Med.

[34] Sterne JAC, Egger M (2001). Funnel plots for detecting bias in meta-analysis: Guidelines on choice of axis. J Clin Epidemiol.

[35] Duval S, Tweedie R (2000). Trim and fill: A simple funnel-plot-based method of testing and adjusting for publication bias in meta-analysis. Biometrics.

[36] Harbord RM, Higgins JPT (2008). Meta-regression in Stata. Stata J.

[37] Sun DW, Zhang D, Tian RH (2020). The underlying changes and predicting role of peripheral blood inflammatory cells in severe COVID-19 patients: A sentinel?. Clin Chim Acta.

[38] Shi Y, Yu X, Zhao H, Wang H, Zhao R, Sheng J (2020). Host susceptibility to severe COVID-19 and establishment of a host risk score: Findings of 487 cases outside Wuhan. Crit Care.

[39] Liu J, Li S, Liu J (2020). Longitudinal characteristics of lymphocyte responses and cytokine profiles in the peripheral blood of SARS-CoV-2 infected patients. EBioMedicine.

[40] Liu D, Wang Y, Wang J (2020). Characteristics and outcomes of a sample of patients with COVID-19 identified through social media in Wuhan, China: Observational study. J Med Internet Res.

[41] Li YK, Peng S, Li LQ (2020). Clinical and Transmission Characteristics of Covid-19 — A Retrospective Study of 25 Cases from a Single Thoracic Surgery Department. Curr Med Sci.

[42] Huang C, Wang Y, Li X (2020). Clinical features of patients infected with 2019 novel coronavirus in Wuhan, China. Lancet.

[43] Guan W, Ni Z, Hu Y (2020). Clinical characteristics of coronavirus disease 2019 in China. N Engl J Med.

[44] Chen L, Yu J, He W (2020). Risk factors for death in 1859 subjects with COVID-19. Leukemia.

[45] Zhou F, Yu T, Du R (2020). Clinical course and risk factors for mortality of adult inpatients with COVID-19 in Wuhan, China: a retrospective cohort study. Lancet.

[46] Zheng Y, Xiong C, Liu Y (2020). Epidemiological and clinical characteristics analysis of COVID-19 in the surrounding areas of Wuhan, Hubei Province in 2020. Pharmacol Res.

[47] Zhang JJ, Dong X, Cao YY (2020). Clinical characteristics of 140 patients infected with SARS-CoV-2 in Wuhan, China. Allergy.

[48] Zhan T, Liu M, Tang Y (2020). Retrospective analysis of clinical characteristics of 405 patients with COVID-19. J Int Med Res.

[49] Yu Q, Wang Y, Huang S (2020). Multicenter cohort study demonstrates more consolidation in upper lungs on initial CT increases the risk of adverse clinical outcome in COVID-19 patients. Theranostics.

[50] Wang R, Pan M, Zhang X (2020). Epidemiological and clinical features of 125 Hospitalized Patients with COVID-19 in Fuyang, Anhui, China.

[51] Parra-Bracamonte GM, Lopez-Villalobos N, Parra-Bracamonte FE (2020). Clinical characteristics and risk factors for mortality of patients with COVID-19 in a large data set from Mexico. Ann Epidemiol.

[52] Kishaba T, Maeda A, Nabeya D, Nagano H (2020). Potential Predictors of Poor Prognosis among Critical COVID-19 Pneumonia Patients Requiring Tracheal Intubation. Tohoku J Exp Med.

[53] Borobia A, Carcas A, Arnalich F (2020). A Cohort of Patients with COVID-19 in a Major Teaching Hospital in Europe. J Clin Med.

[54] Torres-Macho J, Ryan P, Valencia J (2020). The PANDEMYC Score. An Easily Applicable and Interpretable Model for Predicting Mortality Associated With COVID-19. J Clin Med.

[55] Bellan M, Patti G, Hayden E (2020). Fatality rate and predictors of mortality in an Italian cohort of hospitalized COVID-19 patients. Sci Rep.

[56] Di Castelnuovo A, Bonaccio M, Costanzo S (2020). Common cardiovascular risk factors and in-hospital mortality in 3,894 patients with COVID-19: survival analysis and machine learning-based findings from the multicentre Italian CORIST Study. Nutr Metab Cardiovasc Dis.

[57] Zinellu A, Arru F, De Vito A (2020). The De Ritis ratio as prognostic biomarker of in-hospital mortality in COVID-19 patients. Eur J Clin Invest.

[58] CDC COVID-19 Response Team (2020). Preliminary Estimates of the Prevalence of Selected Underlying Health Conditions Among Patients with Coronavirus Disease 2019 — United States, February 1;2–March 28, 2020. MMWR Morb Mortal Wkly Rep.

[59] Langer-Gould A, Smith JB, Gonzales EG (2020). Early identification of COVID-19 cytokine storm and treatment with anakinra or tocilizumab. Int J Infect Dis.

[60] Grechukhina O, Greenberg V, Lundsberg LS (2020). Coronavirus disease 2019 pregnancy outcomes in a racially and ethnically diverse population. Am J Obstet Gynecol MFM.

[61] Gu T, Mack JA, Salvatore M COVID-19 outcomes, risk factors and associations by race: a comprehensive analysis using electronic health records data in Michigan Medicine. medRxiv.

[62] Kalligeros M, Shehadeh F, Mylona EK (2020). Association of Obesity with Disease Severity Among Patients with Coronavirus Disease 2019. Obesity.

[63] Monteiro AC, Suri R, Emeruwa IO Obesity and Smoking as Risk Factors for Invasive Mechanical Ventilation in COVID-19: a Retrospective, Observational Cohort Study. medRxiv.

[64] Zhao Z, Chen A, Hou W (2020). Prediction model and risk scores of ICU admission and mortality in COVID-19. PLoS One.

[65] Brenner EJ, Ungaro RC, Gearry RB (2020). Corticosteroids, But Not TNF Antagonists, Are Associated With Adverse COVID-19 Outcomes in Patients With Inflammatory Bowel Diseases: Results From an International Registry. Gastroenterology.

[66] Chand S, Kapoor S, Orsi D (2020). COVID-19-Associated Critical Illness—Report of the First 300 Patients Admitted to Intensive Care Units at a New York City Medical Center. J Intensive Care Med.

[67] Dashti H, Roche E, Bates D, Mora S, Demler O SARS2 simplified scores to estimate risk of hospitalization and death among patients with COVID-19. medRxiv.

[68] Klang E, Kassim G, Soffer S, Freeman R, Levin MA, Reich DL (2020). Severe Obesity as an Independent Risk Factor for COVID-19 Mortality in Hospitalized Patients Younger than 50. Obesity.

[69] Bauer CMT, Morissette MC, Stämpfli MR (2013). The influence of cigarette smoking on viral infections: Translating bench science to impact COPD pathogenesis and acute exacerbations of COPD clinically. Chest.

[70] Farsalinos K, Barbouni A, Poulas K, Polosa R, Caponnetto P, Niaura R (2020). Current smoking, former smoking, and adverse outcome among hospitalized COVID-19 patients: a systematic review and meta-analysis. Ther Adv Chronic Dis.

[71] Emami A, Javanmardi F, Pirbonyeh N, Akbari A (2020). Prevalence of Underlying Diseases in Hospitalized Patients with COVID-19: a Systematic Review and Meta-Analysis. Arch Acad Emerg Med.

[72] Singh AK, Gillies CL, Singh R (2020). Prevalence of co-morbidities and their association with mortality in patients with COVID-19: A systematic review and meta-analysis. Diabetes Obes Metab.

[73] Huang Y, Lu Y, Huang YM (2020). Obesity in patients with COVID-19: a systematic review and meta-analysis. Metabolism.

[74] Wang Y, Ao G, Qi X, Xie B (2020). The association between COVID-19 and asthma: A systematic review and meta-analysis. Clin Exp Allergy.

