# Supplementary material for: Smoking and risk of negative outcomes among COVID-19 patients: A systematic review and meta-analysis
Source: Tob Induc Dis. 2021 Feb 4;19:09. doi: 10.18332/tid/132411 (PMC7857247; doi:10.18332/tid/132411)
Supplement: Supplementary file 1 [file TID-19-09-s1.pdf]

Smoking and risk of negative outcomes among COVID-19 patients: A systematic review and meta-analysis

Adinat Umnuaypornlert, Sukrit Kanchanasurakit, Don Eliseo III Lucero-Prisno, Surasak Saokaew

Supplementary data

Table

Table S1. Search algorithms.

Table S2. Risk of bias assessment of cohort 40 studies included in the meta-analysis by the Newcastle-Ottawa Scale.

Table S3. Description of outcomes in studies included in meta-analysis.

Table S4. Description of participants of included studies.

Table S5. Meta-regression analysis of the heterogeneous findings

Table S6. Excluded studies

Figure

Figure S1. Funnel plot of included studies (Severity; all age group, and ≤ 65 years old)

Figure S2. The graph from Egger's test of included studies in the meta-analysis (Severity; all age group, and ≤ 65 years old)

Figure S3. Funnel plot of included studies (Dead; all age group)

Figure S4. The graph from Egger's test of included studies in the meta-analysis (Dead; all age group)

Figure S5. Funnel plot of included studies (Dead; ≤65 years old)

Figure S6. The graph from Egger's test of included studies in the meta-analysis (Dead; ≤65 years old)

Tables

Table S1. Search algorithms

Database	No	Step search algorithm	Items found
Pubmed	#1	Search tobacco	129717
	#2	Search smok*	323837
	#3	Search (smok*) OR tobacco	377822
	#4	Search covid	8886
	#5	Search coronavirus	20634
	#6	Search sars cov	7399
	#7	Search ((sars cov) OR coronavirus) OR covid	17717
	#8	Search (((smok*) OR tobacco)) AND (((sars cov) OR coronavirus) OR covid)	833
Cochran	#1	covid	4
	#2	coronavirus	1
	#3	sars cov	3
	#4	#1 or #2 or #3	10
	#5	tobacco	98
	#6	smoking	118
	#7	#5 or #6	210
	#8	#4 and #7	51
Science Direct	#1	covid	4514
	#2	coronavirus	17501
	#3	sars cov	6721
	#4	#1 or #2 or #3	2110
	#5	tobacco	247618
	#6	smoking	455334
	#7	#5 or #6	91948
	#8	#4 and #7	102
Google scholar	#1	covid	37111
	#2	coronavirus	21090
	#3	sars cov	16882
	#4	#1 or #2 or #3	5290
	#5	tobacco	21900
	#6	smoking	33011
	#7	#5 or #6	5420
	#8	#4 and #7	28

Database	No	Step search algorithm	Items found
EMBASE	#1	covid	47101
	#2	coronavirus	22390
	#3	sars cov	16511
	#4	#1 or #2 or #3	3260
	#5	tobacco	21120
	#6	smoking	31521
	#7	#5 or #6	4520
	#8	#4 and #7	234

Table S2. Risk of bias assessment of 40 studies included in the meta-analysis by the Newcastle-Ottawa Scale

**Author (Year)**	**Adequacy selection of case-control**				**Comparability of studies**		**Outcome assessment**			**Total NOS score**
	Is the case definition adequate?	Representativeness of the cases	Selection of Controls	Definition of Controls	Study control for age and sex	Additional factors; controlled for ≥ 2 variables including DM, HT, CVD, ACEIs/ ARBs	Ascertainment of exposure	Same method of ascertainment for cases and controls	Non-Response rate	
Almazeedi S. (2020)	֍	֍	֍	֍	֍	-	֍	֍	-	7/9
Bahl A. (2020)	֍	֍	֍	֍	-	-	֍	֍	-	6/9
Bellan M. (2020)	֍	֍	֍	֍	֍	-	֍	֍	-	7/9
Bi X. (2020)	֍	֍	֍	֍	-	-	֍	֍	-	6/9
Borobia A. M. (2020)	֍	֍	֍	-	֍	-	֍	-	֍	6/9
Brenner E.J. (2020)	֍	֍	֍	֍	-	-	֍	֍	-	6/9
Castelnuovo A.D. (2020)	֍	֍	֍	֍	֍	-	֍	֍	-	7/9
CDC COVID19	-	֍	֍	֍	-	-	֍	֍	-	5/9

Author (Year)	Adequacy selection of case-control				Comparability of studies		Outcome assessment			Total NOS score
	Is the case definition adequate?	Representativeness of the cases	Selection of Controls	Definition of Controls	Study control for age and sex	Additional factors; controlled for ≥ 2 variables including DM, HT, CVD, ACEIs/ ARBs	Ascertainment of exposure	Same method of ascertainment for cases and controls	Non-Response rate	
response team (2020)										
Chand S. (2020)	֍	֍	֍	֍	-	-	֍	֍	-	6/9
Chen L. (2020)	֍	֍	֍	֍	֍	֍	֍	֍	-	8/9
Dashti H. (2020)	֍	֍	֍	֍	-	-	֍	֍	-	6/9
Grechukhina O. (2020)	֍	֍	֍	֍	-	-	֍	֍	֍	7/9
Gu T. (2020)	֍	֍	֍	֍	֍	֍	֍	֍	-	8/9
Guan W (2020)	֍	֍	֍	֍	-	-	֍	֍	-	6/9
Hu L. (2020)	֍	֍	֍	֍	-	-	֍	֍	-	6/9
Huang C (2020)	֍	֍	֍	֍	-	-	֍	֍	-	6/9
Kalligeros	֍	֍	֍	֍	֍	֍	֍	֍	-	8/9

Author (Year)	Adequacy selection of case-control				Comparability of studies		Outcome assessment			Total NOS score
	Is the case definition adequate?	Representativeness of the cases	Selection of Controls	Definition of Controls	Study control for age and sex	Additional factors; controlled for ≥ 2 variables including DM, HT, CVD, ACEIs/ ARBs	Ascertainment of exposure	Same method of ascertainment for cases and controls	Non-Response rate	
M. (2020)										
Kim E.S. (2020)	-	֍	֍	֍	-	-	֍	֍	-	5/9
Kishaba T. (2020)	֍	-	֍	֍	-	-	֍	֍	-	6/9
Klang E. (2020)	֍	֍	֍	֍	֍	֍	֍	֍	-	8/9
Langer-Gould A. (2020)	֍	֍	֍	֍	֍	-	֍	֍	-	7/9
Li X. (2020)	֍	֍	֍	֍	-	-	֍	֍	-	6/9
Li YK. (2020)	֍	֍	֍	֍	-	-	֍	֍	-	6/9
Liu D. (2020)	-	-	֍	֍	֍	-	֍	֍	֍	
Liu J. (2020)	֍	֍	֍	֍	-	-	֍	֍	-	6/9
Monteiro	֍	֍	֍	֍	-	-	֍	֍	-	6/9

Author (Year)	Adequacy selection of case-control				Comparability of studies		Outcome assessment			Total NOS score
	Is the case definition adequate?	Representativeness of the cases	Selection of Controls	Definition of Controls	Study control for age and sex	Additional factors; controlled for ≥ 2 variables including DM, HT, CVD, ACEIs/ ARBs	Ascertainment of exposure	Same method of ascertainment for cases and controls	Non-Response rate	
A.C. (2020)										
Parra-Bracamonte G. M. (2020)	֍	֍	֍	֍	-	-	֍	֍	-	6/9
Shi Y. (2020)	֍	֍	֍	֍	-	-	֍	֍	-	6/9
Sun DW. (2020)	֍	֍	֍	֍	-	-	֍	֍	-	6/9
Torres-Macho J. (2020)	֍	֍	֍	֍	-	-	֍	֍	-	6/9
Wang R. (2020)	֍	֍	֍	֍	-	-	֍	֍	-	6/9
Yang X. (2020)	֍	֍	֍	֍	-	-	֍	֍	-	6/9
Yu Q.	֍	֍	֍	֍	֍	-	֍	֍	-	7/9

**Author (Year)**	**Adequacy selection of case-control**				**Comparability of studies**		**Outcome assessment**			**Total NOS score**
	Is the case definition adequate?	Representativeness of the cases	Selection of Controls	Definition of Controls	Study control for age and sex	Additional factors; controlled for ≥ 2 variables including DM, HT, CVD, ACEIs/ ARBs	Ascertainment of exposure	Same method of ascertainment for cases and controls	Non-Response rate	
(2020)										
Yu T. (2020)	֍	֍	֍	֍	-	-	֍	֍	-	6/9
Zhan T. (2020)	֍	֍	֍	֍	-	-	֍	֍	-	6/9
Zhang JJ. (2020)	֍	֍	֍	֍	-	-	֍	֍	-	6/9
Zhao Z. (2020)	֍	֍	֍	֍	-	-	֍	֍	֍	7/9
Zheng Y. (2020)	֍	֍	֍	֍	-	-	֍	֍	-	6/9
Zhou F. (2020)	֍	֍	֍	֍	-	-	֍	֍	-	6/9
Zinellu A. (2020)	֍	֍	֍	֍	֍	-	֍	֍	-	7/9

Table S3. Description of outcomes in studies included in meta-analysis

Author (Year)	Outcomes	Definitions
Almazeedi S. (2020)	ICU/non-ICU	Assessment of Clinical Criteria for Sepsis Using the qSOFA score for sepsis; For the Third International Consensus Definitions for Sepsis and Septic Shock (Sepsis-3)
Bahl A. (2020)	Dead/Alive	-
Bellan M. (2020)	Dead/Alive	-
Bi X. (2020)	Severe/non-severe	Severe having any of the three conditions: respiratory distress, respiratory rate > 30 breaths/min, in resting state, oxygen saturation is less than 93%, PaO2/ FiO2 ≤300 mmHg. Critical having one of the three conditions; shock incidence, respiratory failure and require mechanical ventilation, admission to ICU with other organ function failure.
Borobia A. M. (2020)	Dead/alive	-
Brenner E.J. (2020)	Dead/alive	-
Castelnuovo A.D. (2020)	Dead/Alive	-
CDC COVID19 response team (2020)	ICU/non ICU + non hospitalization	-
Chand S. (2020)	Dead/Alive	-
Chen L. (2020)	Dead/Alive	-
Dashti H. (2020)	Dead/Alive	-
Grechukhina (2020)	Severe/non-severe	Signs of severe pneumonia, ie, respiratory rate of 30/minute or more, blood oxygen saturation of < 95% {the threshold for O2 supplementation in pregnancy], severe

Author (Year)	Outcomes	Definitions
		respiratory distress, and critical (acute respiratory distress syndrome, sepsis, or septic shock)
Gu T. (2020)	ICU/non-ICU	-
Guan W (2020)	Severe/non-severe	Validated definition includes either one major criterion or three or more minor criteria<br>**Minor criteria**<br>Respiratory rate > 30 breaths/min<br>PaO2/FIO2 ratio < 250<br>Multilobar infiltrates<br>Confusion/disorientation<br>Uremia (blood urea nitrogen level > 20 mg/dl)<br>Leukopenia* (white blood cell count , 4,000 cells/ml)<br>Thrombocytopenia (platelet count , 100,000/ml)<br>Hypothermia (core temperature , 368C)<br>Hypotension requiring aggressive fluid resuscitation<br>**Major criteria**<br>Septic shock with need for vasopressors<br>Respiratory failure requiring mechanical ventilation<br>*Due to infection alone (i.e., not chemotherapy induced).
Hu L. (2020)	Severe/non-severe	Diagnosis complied with the WHO interim guidance and the guidelines of COVID-19 diagnosis and treatment trial (5th edition), by the National Commission of the people's Republic of China.
Huang C (2020)	ICU/non-ICU	-

Author (Year)	Outcomes	Definitions
Kalligeros M. (2020)	ICU/non-ICU	-
Kim E.S. (2020)	Severe/non-severe	Need oxygen supplement therapy
Kishaba T. (2020)	Dead/Alive	-
Klang E. (2020)	Dead/Alive	-
Langer-Gould A. (2020)	Dead/Alive	-
Li X. (2020)	Severe, critically ill/non-severe	Validated definition includes either one major criterion or three or more minor criteria<br>**Minor criteria**<br>Respiratory rate > 30 breaths/min<br>PaO2/FIO2 ratio < 250<br>Multilobar infiltrates<br>Confusion/disorientation<br>Uremia (blood urea nitrogen level > 20 mg/dl)<br>Leukopenia* (white blood cell count , 4,000 cells/ml)<br>Thrombocytopenia (platelet count , 100,000/ml)<br>Hypothermia (core temperature , 368C)<br>Hypotension requiring aggressive fluid resuscitation<br>**Major criteria**<br>Septic shock with need for vasopressors<br>Respiratory failure requiring mechanical ventilation<br>*Due to infection alone (i.e., not chemotherapy induced).
Li YK. (2020)	Severe/non-severe	Severe stage, 1 of the following criteria existed: (a) shortness of breath, respiratory rate

Author (Year)	Outcomes	Definitions
		≥30 times/min; (b) in resting state, oxygen saturation is less than 93%; (c) PaO2/ FiO2 ≤300 mmHg. CT imaging showed significant lesion progression >50% within 24 to 48 h; (d) Respiratory failure requiring mechanical ventilation; (e) Shock; (f) Complicated with other organ failure requiring ICU care.
Liu D. (2020)	Dead/Alive	-
Liu J. (2020)	Severe/mild	severe patients have 1 of the following conditions; (1) shortness of breath, respiratory rate ≥ 30 times/min (2) O2 sat resting rate ≤ 93% or (3) PaO2/FiO2 ≤ 300 mmHg
Monteiro A.C. (2020)	ICU/non-ICU	Any admission to the ICU or any intubation for respiratory failure, regardless of duration, was included in the rate of ICU admissions and intubations, respectively.
Parra-Bracamonte G. M. (2020)	Dead/alive	-
Shi Y. (2020)	Severe/mild	-
Sun DW. (2020)	Severe/non-severe	Severe patients have 1 of the following conditions; (1) shortness of breath, respiratory rate ≥ 30 times/min (2) O2 sat resting rate ≤ 93% or (3) PaO2/FiO2 ≤ 300 mmHg. Critically severe type was defined if any of the items was presented: Respiratory failure requiring mechanical ventilation; (e) Shock; (f) Complicated with other organ failure requiring ICU care.
Torres-Macho J. (2020)	Dead/Alive	-
Wang R. (2020)	Critical/non critical	Defined as the general office of national health commission China, version5 , 2020
Yang X. (2020)	Dead/alive	-
Yu Q. (2020)	Severe/non-severe	Admission to ICU, acute respiratory failure, and shock during hospitalization
Yu T. (2020)	with ARDs / without ARDs	Acute respiratory syndrome = O2 index (arterial partial pressure of O2/fraction of inspired

Author (Year)	Outcomes	Definitions
		O2 ≤ 300 mmHg
Zhan T. (2020)	Severe/non-severe	Severity was established based on respiratory functions on admission with one of the below criteria: (a) shortness of breath, respiratory frequency ≥ 30/min, (b) oxygen saturation ≤ 93% at rest and (c) PaO2/FiO2 ≤ 300 mmHg
Zhang JJ. (2020)	Severe/non-severe	Severity was established based on respiratory functions on admission with one of the below criteria: (a) shortness of breath, respiratory frequency ≥ 30/min, (b) oxygen saturation ≤ 93% at rest and (c) PaO2/FiO2 ≤ 300 mmHg
Zhao Z. (2020)	ICU/non-ICU	-
Zheng Y. (2020)	Severe or critical / ordinary	-
Zhou F. (2020)	Dead/alive	-
Zinellu A. (2020)	Dead/alive	-

Table S4. Description of participants of included studies.

Author, Year	Outcomes	Criteria		Age of participants (years old)		Sex of participants			Comorbidity of participants		
		Inclusion	Exclusion	Interventions	Comparators	Sex	Interventions	Comparators	Diseases	Interventions (%)	Comparators (%)
Almazeedi S. (2020)	ICU/non-ICU	All patients admitted to JaberAl-AhmadAl-Sabah hospital in Kuwait, with a diagnosis of COVID-19, based on the World Health Organization (WHO) interim guidance and have been confirmed by laboratory testing using PCR testing,	Patients who had equivocal PCR test results or were suspected cases were excluded from the study.	54.8±11	37.1±16	Male	32 (3.6%)	856 (96.4%)	Hypertension	17 (9.6)	160 (90.4)
									Diabetes mellitus	18 (11.6)	137 (88.4)
									Dyslipidemia	7 (10.8)	58 (89.2)
									Asthma	6 (14.0)	37 (86.0)
									Coronary artery disease/ischemic heart disease	8 (19.5)	33 (80.5)
									Hypothyroidism	3 (12.0)	22 (88.0)
									Cancer	1 (7.1)	13 (92.9)
									Chronic renal disease	3 (27.3)	8 (72.7)
									Cerebrovascular disease	1 (14.3)	6 (85.7)
									Hepatitis	1 (16.7)	5 (83.3)
									Chronic obstructive pulmonary disease	2 (40.0)	3 (60.0)
									Recent surgery (30 days prior to initial presentation)	0 (0.0)	4 (100.0)

Author, Year	Outcomes	Criteria		Age of participants (years old)		Sex of participants			Comorbidity of participants		
		Inclusion	Exclusion	Interventions	Comparators	Sex	Interventions	Comparators	Diseases	Interventions (%)	Comparators (%)
		between February 24th 2020 and the study's cutoff date of April 20th 2020 were included in the study							Immunodeficiency<br>Other	0 (0.0)<br>5 (15.6)	1(0.0)<br>27 (84.4)
	Dead/alive			55.0±10.1	38.7±15.1	Male	16 (1.8%)	872 (98.2%)	Hypertension<br>Diabetes mellitus<br>Dyslipidemia<br>Asthma<br>Coronary artery disease/ischemic heart disease<br>Hypothyroidism<br>Cancer<br>Chronic renal disease<br>Cerebrovascular disease<br>Hepatitis<br>Chronic obstructive pulmonary disease<br>Recent surgery (30 days prior to initial presentation)	8 (4.5)<br>6 (3.9)<br>3 (4.6)<br>4 (9.3)<br>5 (12.2)<br>1 (4.0)<br>0 (0.0)<br>3 (27.3)<br>0 (0.0)<br>1 (16.7)<br>1 (20.0)<br>0 (0.0)	169 (95.5)<br>149 (96.1)<br>62 (95.4)<br>39 (90.7)<br>36 (87.8)<br>24 (96.0)<br>14 (100.0)<br>8 (72.7)<br>7 (100.0)<br>5 (83.3)<br>4 (80.0)<br>4 (100.0)

Author, Year	Outcomes	Criteria		Age of participants (years old)		Sex of participants			Comorbidity of participants		
		Inclusion	Exclusion	Interventions	Comparators	Sex	Interventions	Comparators	Diseases	Interventions (%)	Comparators (%)
									Immunodeficiency<br>Other	0 (0.0)<br>3 (9.4)	1 (100.0)<br>29 (90.6)
Bahl A. (2020)	Dead/alive	Patients over 18 years of age who were admitted with COVID-19 from March 1st through March 31st 2020 were included	Exclusions consisted of patients who left the hospital against medical advice, transfers to external hospitals, or if hospital course was ongoing beyond April 23,	73	59	Male	180 (55.0%)	590 (52.0%)	Asthma<br>Cancer<br>Coronary artery disease<br>Chronic heart failure<br>Chronic kidney disease<br>Chronic obstructive pulmonary disease<br>Cerebrovascular accident<br>Diabetes mellitus<br>End-stage renal disease<br>Human immunodeficiency virus<br>Hypertension	30 (9.2)<br>44 (13.5)<br>59 (18.0)<br>37 (11.3)<br>33 (10.1)<br>48 (14.7)<br>30 (9.2)<br>127 (38.8)<br>15 (4.6)<br>1 (0.3)<br>200 (61.2)	124 (10.9)<br>110 (9.7)<br>104 (9.2)<br>63 (5.6)<br>42 (3.7)<br>84 (7.4)<br>58 (5.1)<br>303 (26.7)<br>21 (1.9)<br>7 (0.6)<br>551 (48.6)

Author, Year	Outcomes	Criteria		Age of participants (years old)		Sex of participants			Comorbidity of participants		
		Inclusion	Exclusion	Interventions	Comparators	Sex	Interventions	Comparators	Diseases	Interventions (%)	Comparators (%)
			2020						Obstructive Sleep Apnea<br>Pulmonary Hypertension<br>Venous thromboembolism	29 (8.9)<br>6 (1.8)<br>29 (8.9)	90 (7.9)<br>6 (0.5)<br>52 (4.6)
Bellan M. (2020)	Dead/alive	From hospital administrative data revision, we selected all consecutive patients older than 18 years of age, admitted to the hospital afer Emergency Room evaluation, with a	-	77	65	Male	74	165	Arterial Hypertension<br>Diabetes<br>Ischemic cardiopathy<br>Obesity<br>COPD<br>Active Malignancy<br>Chronic liver disease<br>Autoimmune disease<br>Atrial fibrillation<br>Interstitial lung disease<br>Dementia	83<br>31<br>24<br>28<br>21<br>14<br>18<br>7<br>5<br>19<br>4	138<br>58<br>30<br>32<br>39<br>23<br>15<br>6<br>6<br>26<br>6

Author, Year	Outcomes	Criteria		Age of participants (years old)		Sex of participants			Comorbidity of participants		
		Inclusion	Exclusion	Interventions	Comparators	Sex	Interventions	Comparators	Diseases	Interventions (%)	Comparators (%)
		confrmed diagnosis of SARS-CoV-2 infection by reverse-transcriptase polymerase chain reaction (RT-PCR) of a nasopharyngeal swab, between 1st March 2020 and 28th April 2020.							Chronic kidney disease	29<br>28	27<br>22
Bi X. (2020)	Severe/non-severe	One hundred and thirteen patients of COVID-19 were enrolled in Taizhou	-	54	44	Male	13 (59.1%)	51 (56.0%)	-	-	-

Author, Year	Outcomes	Criteria		Age of participants (years old)		Sex of participants			Comorbidity of participants		
		Inclusion	Exclusion	Interventions	Comparators	Sex	Interventions	Comparators	Diseases	Interventions (%)	Comparators (%)
		Public Health Medical Center, Taizhou Hospital, Zhejiang Province, China, from January 23 to February 4, 2020.									
Borobia A.M. (2020)	Dead/alive	Study included 18 years and older, who were hospitalized in the ward of La Paz university hoapital with a diagnosis of covid-19 and	Patients discharged from the emergency department after stay of less than 24 h were not considere	82.5	56	Male	286 (62.2%)	788 (44.6%)	Arterial hypertension<br>Chronic heart disease<br>Diabetes mellitus<br>Rheumatological disease<br>Solid malignant disease<br>Obesity<br>Chronic Kidney	318 (69.1)<br>195 (42.4)<br>157 (34.1)<br>80 (17.4)<br>93 (20.2)<br>66 (14.3)	602 (34.1)<br>234 (13.3)<br>224 (12.7)<br>188 (10.6)<br>159 (9.0)<br>176 (10.0)

Author, Year	Outcomes	Criteria		Age of participants (years old)		Sex of participants			Comorbidity of participants		
		Inclusion	Exclusion	Interventions	Comparators	Sex	Interventions	Comparators	Diseases	Interventions (%)	Comparators (%)
		who either died or were discharged by 19 April. Data of patients were extracted from the electronic prescription system.	d hospitalized and were not included in this analysis.						Disease<br>COPD<br>Other chronic lung disease<br>Hematological malignant disease<br>Asthma<br>Liver disease<br>HIV infection	94 (20.4)<br>65 (14.1)<br>49 (10.7)<br>46 (10.0)<br>17 (3.7)<br>23 (5.0)<br>4 (0.9)	80 (4.5)<br>88 (5.0)<br>94 (5.3)<br>87 (4.9)<br>98 (5.5)<br>66 (3.7)<br>9 (0.5)
renner E.J. (2020)	Dead/alive	We created the Surveillance Epidemiology of Coronavirus Under Research Exclusion for Inflammatory Bowel	-	-	-	Male	11 (4%)	514 (89%)	Any condition<br>Cardiovascular disease (eg, CAD, heart failure, arrhythmia)<br>Diabetes<br>Lung disease (eg, asthma, COPD)<br>Hypertension<br>Cancer<br>History of stroke	192 (36.6)<br>38 (7.2)<br>29 (5.5)<br>44 (8.4)<br>63 (12.0)<br>10 (1.9)<br>4 (0.8)	

Author, Year	Outcomes	Criteria		Age of participants (years old)		Sex of participants			Comorbidity of participants		
		Inclusion	Exclusion	Interventions	Comparators	Sex	Interventions	Comparators	Diseases	Interventions (%)	Comparators (%)
		Disease (SECURE-IBD) database to monitor outcomes of COVID-19 occurring in pediatric and adult patients with IBD. SECURE-IBD							Chronic renal disease (eg, CKD)<br>Chronic liver disease (eg, PSC, NAFLD, cirrhosis)<br>Other	10 (1.9)<br>26 (5.0)<br>53 (10.1)	
Castelnuovo A.D. (2020)	Dead/alive	Data for the present analyses were provided by 30 hospitals distributed throughout Italy. Each hospital provided data	77 Patients were excluded from the analysis because of missing data ()	-	-	Male	469 (19.5%)	-	Hypertension<br>Diabetes<br>Myocardial infarction<br>Heart failure<br>Cancer<br>Lung disease<br>Obesity	461 (23.7)<br>203 (27.5)<br>140 (36.1)<br>165 (38.3)<br>128 (32.1)<br>167 (29.6)<br>69 (18.4)	-

Author, Year	Outcomes	Criteria		Age of participants (years old)		Sex of participants			Comorbidity of participants		
		Inclusion	Exclusion	Interventions	Comparators	Sex	Interventions	Comparators	Diseases	Interventions (%)	Comparators (%)
		from hospitalised adult (18 years of age) patients who all had a positive test result for the SARSCoV-2 virus at any time during their hospitalisation from February 19th to May 23rd, 2020. The follow-up continued through May 29th, 2020.									
CDC COVID19	ICU/non ICU + non	A total of US states and	-	≥19		-	-	-	Diabetes mellitus<br>Chronic lung	784 (10.9)<br>656 (9.2)	

Author, Year	Outcomes	Criteria		Age of participants (years old)		Sex of participants			Comorbidity of participants		
		Inclusion	Exclusion	Interventions	Comparators	Sex	Interventions	Comparators	Diseases	Interventions (%)	Comparators (%)
response team (2020)	hospitalization	territories have reported 122,653 US CPVID-19 cases to CDC, including 7,162 for whom data on underlying health conditions and other known risk factors for severe outcomes from respiratory infections were reported.							disease<br>Cardiovascular disease<br>immunocompromised conditions<br>Chronic renal disease<br>Pregnancy<br>Neurologic disorder, neurodevelopmental, intellectual disability<br>Chronic liver disease<br>Other chronic disease	 647 (9.0)<br>264 (3.7)<br>213 (3.0)<br>143 (2.0)<br>52 (0.7)<br>41 (0.6)<br>1,182 (16.5)	
Chand S. (2020)	Dead/alive	Consecutive adult patients aged 18 years	-	60.6	55.6	Male	60.4 (11.9%)	54.1 (11.7%)	None<br>Cirrhosis<br>Diabetes	22 (14)<br>1 (0.6)<br>73 (46.5)	35 (24.5)<br>1 (0.7)<br>61 (42.7)

Author, Year	Outcomes	Criteria		Age of participants (years old)		Sex of participants			Comorbidity of participants		
		Inclusion	Exclusion	Interventions	Comparators	Sex	Interventions	Comparators	Diseases	Interventions (%)	Comparators (%)
		and older admitted to 9 ICUs within the 3 main hospitals comprising Montefiore Medical Center (Bronx, NY) with documented SARS-CoV-2 infection were identified and their electronic medical records manually reviewed for extraction of							Hypertension<br>Asthma<br>Chronic obstructive pulmonary disease<br>Coronary Artery disease<br>ESRD on hemodialysis<br>CKD<br>HIV<br>Heart Failure with reduced ejection fraction<br>Heart Failure with preserved ejection fraction<br>Cancer	115 (73.2)<br>17 (10.8)<br>11 (7)<br>24 (15.3)<br>12 (7.6)<br>22 (14.0)<br>2 (1.3)<br>11 (7)<br>7 (4.5)<br>13 (8.3)	85 (59.4)<br>22 (15.4)<br>6 (4.2)<br>17 (11.9)<br>4 (2.8)<br>17 (11.9)<br>3 (2.1)<br>5 (3.5)<br>1 (0.7)<br>5 (3.5)

Author, Year	Outcomes	Criteria		Age of participants (years old)		Sex of participants			Comorbidity of participants		
		Inclusion	Exclusion	Interventions	Comparators	Sex	Interventions	Comparators	Diseases	Interventions (%)	Comparators (%)
		relevant demographic and medical data									
Chen L. (2020)	Dead/alive	Between February 4 and February 18, 2020 persons with clinical symptoms and a lung computed tomography (CT) scan consistent with COVID-19 were diagnosed as having COVID-19 without	Subjects in whom we could not confirm SARS-CoV-2-infection by a qRT-PCR, IgM/IgG assay, or either were excluded from the study	-	-	Female	55 (26%)	870 (53%)	Atherosclerotic cardio and cerebro-vascular disease<br>Hypertension<br>Diabetes<br>COPD<br>Cancer<br>Chronic kidney disease<br>Gastro-intestinal disease<br>Auto-immune disease<br>Psychiatric disorders	63 (30)<br>104 (50)<br>59 (28)<br>12 (6)<br>17 (8)<br>20 (10)<br>16 (8)<br>1 (1)<br>1 (1)	205 (12)<br>475 (29)<br>203 (12)<br>49 (3)<br>52 (3)<br>25 (2)<br>82 (5)<br>9 (1)<br>6 (0.5)

Author, Year	Outcomes	Criteria		Age of participants (years old)		Sex of participants			Comorbidity of participants		
		Inclusion	Exclusion	Interventions	Comparators	Sex	Interventions	Comparators	Diseases	Interventions (%)	Comparators (%)
		confirmation of SARS-CoV-2-infection by quantitative reverse transcript polymerase chain reaction (qRT-PCR). After hospitalization subjects were tested by qRT-PCR to confirm the diagnosis and monitor their course. Beginning 4 March, 2020, anti- SARS-									

Author, Year	Outcomes	Criteria		Age of participants (years old)		Sex of participants			Comorbidity of participants		
		Inclusion	Exclusion	Interventions	Comparators	Sex	Interventions	Comparators	Diseases	Interventions (%)	Comparators (%)
		CoV-2 IgM and/or IgG antibodies were assayed at Union Hospital and Wuhan Central Hospital by the centers to confirm the diagnosis and to evaluate suspected cases of COVID-19 which were qRT-PCR-negative									
Dashti H. (2020)	Dead/alive	The list of investigated and recorded	-	78	62	Female	215 (42.2%)	1616 (47.5%)	-	-	-

Author, Year	Outcomes	Criteria		Age of participants (years old)		Sex of participants			Comorbidity of participants		
		Inclusion	Exclusion	Interventions	Comparators	Sex	Interventions	Comparators	Diseases	Interventions (%)	Comparators (%)
		risk markers from medical history of patients varied, which could be due to the complexity and challenges associated with extracting phenotypes from electronic health records (EHR) data were analyzed.									
Grechukhina O.	Severe/non-severe	Subjects were identified	-	30.5	30	Female	6 (-)	135(-)	Pregestational Diabetes	1	6

Author, Year	Outcomes	Criteria		Age of participants (years old)		Sex of participants			Comorbidity of participants		
		Inclusion	Exclusion	Interventions	Comparators	Sex	Interventions	Comparators	Diseases	Interventions (%)	Comparators (%)
(2020)		using electronic health record. Ambulatory and inpatients testing was included. All pregnant and postpartum women with positive SARS-CoV-2 reverse transcription polymerase chain reaction test between March 3, 2020, and May 11, 2020, from 3 Yale New Haven							Chronic hypertension<br>Heart disease<br>Asthma	2<br>0<br>2	12<br>7<br>16

Author, Year	Outcomes	Criteria		Age of participants (years old)		Sex of participants			Comorbidity of participants		
		Inclusion	Exclusion	Interventions	Comparators	Sex	Interventions	Comparators	Diseases	Interventions (%)	Comparators (%)
		Health hospitals.									
Gu T. (2020)	ICU/non-ICU<br>Dead	We extracted the EHR data for patients tested for COVID-19 at the University of Michigan Medicine Health System, also known as Michigan Medicine (MM), from March 10, 2020 to April 22, 2020. To understand how selection bias factored	-	47		Male	138(60.8%)	-	-	-	-

Author, Year	Outcomes	Criteria		Age of participants (years old)		Sex of participants			Comorbidity of participants		
		Inclusion	Exclusion	Interventions	Comparators	Sex	Interventions	Comparators	Diseases	Interventions (%)	Comparators (%)
		into our sample, in addition to comparing COVID-19 positive patients with those testing negative, we created two sets of controls from the MM database.									
Guan W (2020)	Severe/non-severe	The medical records for hospitalized patients and outpatients with Laboratory	-	52	45	Female	73 (42.2%)	386 (41.8%)	Any<br>Chronic obstructive pulmonary disease<br>Diabetes<br>Hypertension<br>Coronary heart	67 (38.7)<br>6 (3.5)<br>28 (16.2)<br>41 (23.7)	194 (21.0)<br>6 (0.6)<br>53 (5.7)<br>124 (13.4)

Author, Year	Outcomes	Criteria		Age of participants (years old)		Sex of participants			Comorbidity of participants		
		Inclusion	Exclusion	Interventions	Comparators	Sex	Interventions	Comparators	Diseases	Interventions (%)	Comparators (%)
		confirmed COVID-19 were reviewed							disease<br>Cerebrovascular disease<br>Hepatitis B infection<br>Cancer<br>Chronic renal disease<br>Immunodeficiency	10 (5.8)<br>4 (2.3)<br>1 (0.6)<br>3 (1.7)<br>3 (1.7)<br>0 (0.0)	17 (1.8)<br>11 (1.2)<br>22 (2.4)<br>7 (0.8)<br>5 (0.5)<br>2 (0.2)
Hu L. (2020)	Severe/non-severe	We analyzed the initial clinical presentation and baseline laboratory test results, as well as clinical course, of 323 hospitalized patients with	-	67	56	Male	91 (53.8%)	75 (49.7%)	Cirrhosis<br>Hypertension<br>Diabetes<br>Malignancy<br>Cerebrovascular disease<br>Chronic obstructive pulmonary disease<br>Chronic kidney disease<br>Chronic liver	0 (0)<br>66 (38.5)<br>33 (19.18)<br>5 (2.90)<br>3 (1.74)<br>6 (1.48)<br>3 (1.74)	3 (2)<br>39 (25.8)<br>14 (9.3)<br>0 (0)<br>4 (2.6)<br>0 (0)<br>4 (2.6)

Author, Year	Outcomes	Criteria		Age of participants (years old)		Sex of participants			Comorbidity of participants		
		Inclusion	Exclusion	Interventions	Comparators	Sex	Interventions	Comparators	Diseases	Interventions (%)	Comparators (%)
		COVID-19 in Wuhan, to identify risk factors associated with clinical outcomes for improving management guidelines.							disease<br>Cardiovascular and cerebrovascular disease<br>Digestive system disease<br>Endocrine system disease<br>Nervous system disease<br>Respiratory system disease	2 (1.16)<br>33 (19.18)<br>14 (8.14)<br>11 (6.40)<br>5 (2.90)<br>21 (12.21)	3 (2)<br>8 (5.3)<br>8 (5.3)<br>4 (2.6)<br>5 (3.3)<br>8 (5.3)
Huang C (2020)	Severe/non-severe	All patients with suspected 2019-nCoV were admitted to designated hospital in Wuhan.	-	49	49	Male	11 (85%)	19 (68%)	Diabetes mellitus<br>Hypertension<br>Cardiovascular disease<br>Chronic obstructive pulmonary disease<br>Malignancy	1 (8)<br>2 (15)<br>3 (23)<br>1 (8)	7 (25)<br>4 (14)<br>3 (11)<br>0 (0.0)

Author, Year	Outcomes	Criteria		Age of participants (years old)		Sex of participants			Comorbidity of participants		
		Inclusion	Exclusion	Interventions	Comparators	Sex	Interventions	Comparators	Diseases	Interventions (%)	Comparators (%)
		Infection confirmed by real-time RT-PCR and next generation sequencing.							Chronic liver disease	0 (0.0)<br>0 (0.0)	1 (4)<br>1 (4)
Kalligeros M. (2020)	ICU/non-ICU	All consecutive adult (≥18 years old) patients who had a laboratory-confirmed (using a reverse transcriptase–polymerase chain reaction assay) SARS-CoV-2 infection and	-	61.5	57	Male	29 (65.9%)	34 (57.6%)	Cancer<br>Chronic renal<br>Cirrhosis<br>Diabetes<br>Heart disease<br>Hypertension<br>Lung disease<br>Transplant	6 (13.6)<br>4 (9.1)<br>0 (0)<br>21 (47.7)<br>14 (31.8)<br>31 (70.4)<br>11 (25.0)<br>1 (2.2)	3 (5.0)<br>7 (11.8)<br>3 (5.0)<br>17 (28.8)<br>11 (18.6)<br>35 (59.3)<br>9 (15.2)<br>1 (1.6)

Author, Year	Outcomes	Criteria		Age of participants (years old)		Sex of participants			Comorbidity of participants		
		Inclusion	Exclusion	Interventions	Comparators	Sex	Interventions	Comparators	Diseases	Interventions (%)	Comparators (%)
		who were admitted to Rhode Island Hospital, The Miriam Hospital, or Newport Hospital in Rhode Island between February 17 and April 5, 2020, were considered eligible for inclusion									
Kim E.S. (2020)	Severe/non-severe	28 hospitalized patients with confirmed COVID-19. All cases	-	42.6±13.4		Male	15 (53.6)		Diabetes mellitus without complication<br>Asthma<br>Liver disease, mild<br>Malignancy	2 (7.1)<br>1 (3.6)<br>1 (3.6)<br>1 (3.6)	

Author, Year	Outcomes	Criteria		Age of participants (years old)		Sex of participants			Comorbidity of participants		
		Inclusion	Exclusion	Interventions	Comparators	Sex	Interventions	Comparators	Diseases	Interventions (%)	Comparators (%)
		confirmed by real-time polymerase chain reaction.							Obesity (Body mass index > $30kg/m^2$)	5 (17.9)	
Kishaba T. (2020)	Dead/alive	Data from critical COVID-19 pneumonia patients at the Okinawa Chubu Hospital who required mechanical ventilation were used.	-	75	73	Male	2	3	-	-	-
Klang E. (2020)	Dead/alive	We retrospective analyzed data (in younger or	-	≤ 50		Male	45 (75.0%)	352 (68.8%)	Coronary artery disease<br>Heart failure<br>Hypertension	6 (10)<br>10 (16.7)<br>24 (40.0)	26 (5.1)<br>25 (4.9)<br>151 (29.5)

Author, Year	Outcomes	Criteria		Age of participants (years old)		Sex of participants			Comorbidity of participants		
		Inclusion	Exclusion	Interventions	Comparators	Sex	Interventions	Comparators	Diseases	Interventions (%)	Comparators (%)
		older than 50) from a large academic hospital system in New York city between March 1st and May 17th, 2020. Data were extracted from electronics medical records.							Diabetes<br>Hyperlipidemia<br>Chronic kidney disease<br>Cancer	24 (40.0)<br>11 (18.3)<br>17 (28.3)<br>9 (15.0)	129 (25.2)<br>63 (12.3)<br>53 (10.4)<br>30 (5.9)
Langer-Gould A. (2020)	Dead/alive	Patients treated with tocilizumab or anakinra were confirmed by electronic databases.	Patients were excluded if they received tocilizumab or	61.9	58.1	Male	27 (81.8)	37 (77.1)	Obesity<br>Diabetes<br>Hypertension<br>Asthma/COPD	19<br>18<br>26<br>2	36<br>27<br>32<br>7

Author, Year	Outcomes	Criteria		Age of participants (years old)		Sex of participants			Comorbidity of participants		
		Inclusion	Exclusion	Interventions	Comparators	Sex	Interventions	Comparators	Diseases	Interventions (%)	Comparators (%)
		COVID19-CS was defined clinically by increasing O2 requirements and bilateral infiltrates on chest X-ray or CT.	anakinra for other indication, the dose and/or duration of treatment were not inadequet and not related to adverse even.								
Li X. (2020)	Severe/non-severe	All case with COVID-19 enrolled in this study were diagnosed on the basis of WHO interim		65	56	Male	153(56.9%)	126 (45.2%)	Chronic obstructive pulmonary disease<br>Asthma<br>Tuberculosis<br>Diabetes mellitus<br>Hypertension<br>Coronary heart	13 (4.8)<br>3 (1.1)<br>4 (1.5)<br>52 (19.3)<br>104 (38.7)<br>28 (10.4)	4 (1.4)<br>2 (0.7)<br>5 (1.8)<br>31 (11.1)<br>62 (22.2)<br>6 (2.2)

Author, Year	Outcomes	Criteria		Age of participants (years old)		Sex of participants			Comorbidity of participants		
		Inclusion	Exclusion	Interventions	Comparators	Sex	Interventions	Comparators	Diseases	Interventions (%)	Comparators (%)
		guidance and the diagnostic and treatment guideline for COVID-19 by the Chinese National Health Committee. (version 5)							disease<br>Hepatitis B<br>Chronic kidney disease<br>Tumor	 2 (0.7)<br>6 (2.2)<br>14 (5.5)	 3 (1.1)<br>4 (1.4)<br>10 (3.9)
Li YK. (2020)	Severe/non-severe	25 cases of COVID-19 (including; 13 hospitalized patients and 12 health care staff) were enrolled in the study. All of them had traceable positive	-	51		Male	6 (66.67%)	6 (37.5%)	Hypertension<br>Diabetes<br>Chronic obstructive pulmonary disease<br>Coronary heart disease	1<br>1<br>4<br>1	1<br>0<br>1<br>3
	Dead/alive						4 (80.0%)	8 (40%)	Hypertension<br>Diabetes	1<br>1	1<br>0

Author, Year	Outcomes	Criteria		Age of participants (years old)		Sex of participants			Comorbidity of participants		
		Inclusion	Exclusion	Interventions	Comparators	Sex	Interventions	Comparators	Diseases	Interventions (%)	Comparators (%)
		exposure history, with a spatiotemporal correlation of close contact and health care staff.							Chronic obstructive pulmonary disease<br>Coronary heart disease	3<br>1	2<br>3
Liu D. (2020)	Dead/alive	We carefully monitored reports of patients infected with COVID-19 on Weibo between January 20 and February 15, 2020, and confirmed case by the follow up	-	72	61	Female	31 (37.3)	243 (47.6)	Hypertension<br>Diabetes<br>Chronic heart disease<br>Chronic lung disease<br>Cerebrovascular disease<br>Chronic kidney disease<br>Chronic liver disease<br>Chronic respiratory disease	8 (9.8)<br>8 (9.8)<br>9 (11.0)<br>3 (3.7)<br>2 (2.4)<br>2 (2.4)<br>14 (2.4)<br>1 (1.2)	79 (15.5)<br>49 (9.6)<br>61 (12.0)<br>22 (4.3)<br>13 (2.5)<br>26 (5.1)<br>0 0()<br>11 (2.2)

Author, Year	Outcomes	Criteria		Age of participants (years old)		Sex of participants			Comorbidity of participants		
		Inclusion	Exclusion	Interventions	Comparators	Sex	Interventions	Comparators	Diseases	Interventions (%)	Comparators (%)
		telephone call. 1 month later, we checked the clinical outcomes of patients and other acquired information. Only patients who had a defined outcome (died or recovered) were included in the study.							Cancer	1 (1.2)	16 (3.1)
Liu J. (2020)	Severe/non-severe	40 confirmed COVID-19 were enrolled. All medical records information was obtained.	-	59.7±10.1	43.2±13.9	Male	7 (53.8%)	8 (29.6%)	-	-	-

Author, Year	Outcomes	Criteria		Age of participants (years old)		Sex of participants			Comorbidity of participants		
		Inclusion	Exclusion	Interventions	Comparators	Sex	Interventions	Comparators	Diseases	Interventions (%)	Comparators (%)
Monteiro A.C. (2020)	Severe/non-severe	Hospitalized patients at RR-UCLA and SM-UCLA > 18 years old with positive SARS-CoV-2 PCR testing from either nasal swab or mini-bronchoalveolar lavage (BAL) testing were included. Data for patients who tested positive for SARS-CoV-2 was manually	-	58	64	Male	19 (68%)	55 (65%)	Obesity<br>Hypertension<br>Diabetes<br>Chronic obstructive pulmonary disease<br>Coronary artery disease<br>Cancer<br>Asthma<br>Atrial fibrillation<br>Chronic kidney disease<br>Transplant recipient	17 (61)<br>17 (61)<br>19 (68)<br>2 (7)<br>3 (11)<br>2 (7)<br>4 (14)<br>1 (4)<br>3 (11)<br>1 (4)	23 (27)<br>39 (46)<br>53 (63)<br>4 (5)<br>14 (17)<br>13 (15)<br>9 (11)<br>10 (12)<br>16 (19)<br>6 (7)

**Author, Year**	**Outcomes**	**Criteria**		**Age of participants (years old)**		**Sex of participants**			**Comorbidity of participants**		
		Inclusion	Exclusion	Interventions	Comparators	Sex	Interventions	Comparators	Diseases	Interventions (%)	Comparators (%)
		extracted from the electronic health record and included in a database.									
Parra-Bracamonte G. M. (2020)	Dead/alive	Information from 475 monitoring units all along the country from the public and private health sectors. Positive cases were extracted and edited. Data from 331,298 patients diagnosed positively to	-	-	-	Male	25015 (65.3%)	153137 (52.3%)	Hypertension<br>Obesity<br>Diabetes<br>Cardiopathy<br>Chronic obstructive pulmonary disease<br>Asthma<br>Immunosuppressed<br>Chronic kidney disease	16,409 (42.8)<br>9504 (24.8)<br>14,295 (37.3)<br>2037 (5.3)<br>1839 (4.8)<br>777 (2.0)<br>1061 (2.8)<br>2588 (6.8)	49,761 (17.0)<br>53,955 (18.4)<br>39,417 (13.5)<br>5314 (1.8)<br>3619 (1.2)<br>8206 (2.8)<br>3135 (1.1)<br>4307 (1.5)

Author, Year	Outcomes	Criteria		Age of participants (years old)		Sex of participants			Comorbidity of participants		
		Inclusion	Exclusion	Interventions	Comparators	Sex	Interventions	Comparators	Diseases	Interventions (%)	Comparators (%)
		COVID-19, from the first positive case registered on January 13 to July 17, 2020 (database accessed in July 18, 2020), were analyzed									
Shi Y. (2020)	Severe/non-severe	487 patients in Zhejiang Province of China were reviewed medical records, laboratory findings, and pulmonary CT scan with		56	45	Male	36 (73.5%)	223 (50.9%)	Hypertension<br>Diabetes<br>Cardiovascular disease<br>Malignancy<br>Chronic liver disease<br>Chronic renal disease<br>Others	26 (53.1)<br>7 (14.3)<br>4 (8.2)<br>2 (4.1)<br>2 (4.1)<br>2 (4.1)<br>5 (10.2)	73 (16.7)<br>22 (5.0)<br>7 (1.6)<br>3 (0.7)<br>20 (4.6)<br>5 (1.1)<br>27 (6.1)

Author, Year	Outcomes	Criteria		Age of participants (years old)		Sex of participants			Comorbidity of participants		
		Inclusion	Exclusion	Interventions	Comparators	Sex	Interventions	Comparators	Diseases	Interventions (%)	Comparators (%)
		COVID-19.									
Sun DW. (2020)	Severe/non-severe	Patients included in this study were 2019-nCoV positive based on nucleic acid detection, from the intensive care unit (ICU) of Tongji hospital affiliated to Huazhong University of Science and Technology. The primary cohort included 45	-	67	61	Male	24 (53.33%)	5 (41.67%)	-	-	-

Author, Year	Outcomes	Criteria		Age of participants (years old)		Sex of participants			Comorbidity of participants		
		Inclusion	Exclusion	Interventions	Comparators	Sex	Interventions	Comparators	Diseases	Interventions (%)	Comparators (%)
		cases of severe type, who were admitted by our assisting team initially on 10th Feb 2020, serving as study group. 12 cases of no-severe type were admitted into this first-aiding hospital by our team on 8th Mar 2020, serving as control group.									
Torres-Macho J.	Dead/alive	The study population	-	82	63	Male	222 (68.7%)	882 (54%)	Chronic Heart Disease	132 (41.2)	283 (17.8)

Author, Year	Outcomes	Criteria		Age of participants (years old)		Sex of participants			Comorbidity of participants		
		Inclusion	Exclusion	Interventions	Comparators	Sex	Interventions	Comparators	Diseases	Interventions (%)	Comparators (%)
(2020)		comprised all patients admitted to hospital with a confirmed diagnosis of COVID-19 based on a positive result in the SARS-CoV-2 reverse transcriptase-polymerase chain reaction assay between 2nd March and 31st May 2020. Samples were obtained via nasopharyngeal swabs.							Hypertension<br>Chronic<br>Pulmonary Disease<br>Asthma<br>Stage 4 Chronic<br>Kidney Disease<br>Liver Cirrhosis<br>Solid Neoplasm<br>(Active)<br>Hematologic<br>Neoplasm (Active)<br>HIV Infection<br>Obesity<br>Diabetes<br>Dyslipidemia<br>Inflammatory<br>Disease<br>Dementia<br>Malnutrition	226 (70.6)<br>69 (21.8)<br><br><br>18 (5.6)<br>42 (13.2)<br><br>9 (2.8)<br>39 (12.3)<br><br>13 (4)<br><br>0 (0)<br>45 (17.3)<br>99 (31)<br>97 (53.6)<br>25 (7.8)<br><br>37 (11.7)<br>14 (5.2)	765 (48)<br>169 (10.7)<br><br><br>138 (8.7)<br>68 (4.3)<br><br>22 (1.4)<br>42 (2.6)<br><br>21 (1.3)<br><br>11 (0.7)<br>239 (17.8)<br>337 (21.2)<br>347 (33.8)<br>74 (4.7)<br><br>71 (4.5)<br>26 (1.8)

Author, Year	Outcomes	Criteria		Age of participants (years old)		Sex of participants			Comorbidity of participants		
		Inclusion	Exclusion	Interventions	Comparators	Sex	Interventions	Comparators	Diseases	Interventions (%)	Comparators (%)
Wang R. (2020)	Severe/non-severe	Patients who were diagnosed with COVID-19 at No.2 hospital were enrolled in the study. These patients confirmed by a positive result for real-time RT-PCR in the respiratory tract samples.		49.40±13.64	39.47±14.84	Male	16 (64%)	55 (55%)	Cardiovascular disease<br>Digestive system disease<br>Endocrine system disease<br>Rheumatic immune disease<br>Respiratory system disease<br>Hematological system disease<br>Nervous system disease<br>Malignant tumor<br>HIV infection	18 (14.4)<br>8 (6.4)<br>10 (8.0)<br>1 (0.8)<br>2 (1.6)<br>1 (0.8)<br>1 (0.8)<br>1 (0.8)<br>1 (0.8)	
Yang X. (2020)	Dead/alive	Patients were diagnosed as SARS-pneumonia, according to WHO interim	-	64.6	51.9	Male	21 (66%)	14 (70%)	Chronic cardiac disease<br>Chronic pulmonary disease<br>Cerebrovascular disease	3 (9)<br>2 (6)<br>7 (22)	2 (10)<br>2 (10)<br>0

Author, Year	Outcomes	Criteria		Age of participants (years old)		Sex of participants			Comorbidity of participants		
		Inclusion	Exclusion	Interventions	Comparators	Sex	Interventions	Comparators	Diseases	Interventions (%)	Comparators (%)
		guidance and who were critically ill.							Diabetes<br>Malignancy<br>Dementia<br>Malnutrition	7 (22)<br>1 (3)<br>1 (3)<br>0	2 (10)<br>1 (5)<br>0<br>0
Yu Q. (2020)	Severe/non-severe	We enrolled patients with laboratory-confirmed COVID-19 in Jiangsu province between 10 January and 18 February 2020. Laboratory confirmation was based on a positive result on high-throughput	We excluded patients aged < 18 years old or with missing clinical or CT records on admission.	57.2	45.0	Male	41 (64)	183 (51)	Hypertension<br>Coronary heart disease<br>Cardiac dysfunction III-IV<br>Liver dysfunctionb<br>Diabetes<br>Chronic kidney disease<br>Malignant tumor<br>Stoke	20(31)<br>4(6)<br>1(2)<br>1(2)<br>9(14)<br>1(2)<br>1(2)<br>1(2)	50(14)<br>3(1)<br>1(0.3)<br>1(0.3)<br>17(5)<br>3(2)<br>4(1)<br>2(0.6)

Author, Year	Outcomes	Criteria		Age of participants (years old)		Sex of participants			Comorbidity of participants		
		Inclusion	Exclusion	Interventions	Comparators	Sex	Interventions	Comparators	Diseases	Interventions (%)	Comparators (%)
		sequencing or real-time reverse-transcriptase–polymerase-chain-reaction (RT-PCR) assay of nasal and pharyngeal swab specimens.									
Yu T. (2020)	Severe/non-severe	95 infected COVID-19 were enrolled. Diagnostic standard was polymerase chain reaction detection of target genes 2019 n-CoV.	-	45.92	35.73	Male	14 (58.3)	39 (54.9)	-	-	-

Author, Year	Outcomes	Criteria		Age of participants (years old)		Sex of participants			Comorbidity of participants		
		Inclusion	Exclusion	Interventions	Comparators	Sex	Interventions	Comparators	Diseases	Interventions (%)	Comparators (%)
		Clinical, laboratories, radiologic results, and treatment outcomes data were obtained.									
Zhan T. (2020)	Severe/non-severe	This study included all hospitalized patients who were admitted to the Third Hospital of Wuhan (one of the designated facilities for hospitalization of patients with COVID-	-	64	52	Male	73 (49.3%)	113 (43.9%)	-	-	-

Author, Year	Outcomes	Criteria		Age of participants (years old)		Sex of participants			Comorbidity of participants		
		Inclusion	Exclusion	Interventions	Comparators	Sex	Interventions	Comparators	Diseases	Interventions (%)	Comparators (%)
		19) from 12 January to 8 March 2020 and diag- nosed with COVID-19 according to a pos- itive result of high-throughput sequencing or real-time reverse-transcriptase polymerase chain reaction assay using nasal or pharyngeal swab specimens.									
Zhang JJ.	Severe/non-	All 242	Patients	64	51.5	Male	33 (56.9%)	38 (46.3%)	Hypertension	22 (37.9)	20 (24.4)

Author, Year	Outcomes	Criteria		Age of participants (years old)		Sex of participants			Comorbidity of participants		
		Inclusion	Exclusion	Interventions	Comparators	Sex	Interventions	Comparators	Diseases	Interventions (%)	Comparators (%)
(2020)	severe	hospitalized patients in a hospital of Wuhan, clinically diagnosed as "viral pneumonia" based on their clinical symptoms with typical changes in chest radiology and were living in Wuhan during the outbreak period of COVID-19.	absent of or with negative SARS-CoV-2 test results were excluded from this study.						Diabetes<br>Fatty liver and abnormal liver function<br>Chronic gastritis and gastric ulcer<br>Coronary heart disease<br>Hyperlipidemia<br>Cholelithiasis<br>Arrhythmia<br>Thyroid disease<br>Electrolyte imbalance<br>Urolithiasis<br>Stroke<br>Chronic renal insufficiency<br>Aorta sclerosis<br>Secondary pulmonary tuberculosis	8 (13.8)<br>4 (6.9)<br>2 (3.4)<br>4 (6.9)<br>2 (3.4)<br>4 (6.9)<br>4 (6.9)<br>4 (6.9)<br>4 (6.9)<br>1 (1.7)<br>2 (3.4)<br>2 (3.4)<br>1 (1.7)<br>2 (3.4)	9 (11.0)<br>4 (5.0)<br>5 (6.1)<br>3 (3.7)<br>5 (6.1)<br>2 (2.4)<br>1 (1.2)<br>1 (1.2)<br>0<br>2 (2.4)<br>1 (1.2)<br>0<br>1 (1.2)<br>0

Author, Year	Outcomes	Criteria		Age of participants (years old)		Sex of participants			Comorbidity of participants		
		Inclusion	Exclusion	Interventions	Comparators	Sex	Interventions	Comparators	Diseases	Interventions (%)	Comparators (%)
									Chronic obstructive pulmonary disease	2 (3.4)	0
Zhao Z. (2020)	Severe/non-severe	Hospitalized patients who were diagnosed by positive RT-PCR for SARS-CoV-2 were included in this study.	Patients who were still hospitalized because their outcomes unknown at the time, thus risking grouping them incorrectly. Patients who were younger than 18	60	58	Male	136 (69.7%)	222 (55.8%)	Diabetes<br>Hypertension<br>Asthma<br>COPD<br>Coronary artery disease<br>Heart failure<br>Cancer<br>Immunosuppression<br>Chronic Kidney disease	58 (29.7)<br>96 (49.2)<br>16 (8.2)<br>11 (5.6)<br>22 (11.3)<br>10 (5.1)<br>9 (4.6)<br>12 (6.2)<br>16 (8.2)	104 (26.1)<br>170 (42.7)<br>25 (6.3)<br>25 (6.3)<br>46 (11.6)<br>10 (2.5)<br>25 (6.3)<br>22 (5.5)<br>28 (7.1)
	Dead/alive			77	58	Male	53 (64.6%)	222 (55.8%)	Diabetes<br>Hypertension<br>Asthma<br>COPD<br>Coronary artery disease	25 (30.5)<br>52 (63.4)<br>3 (3.7)<br>15 (18.3)<br>25 (30.5)	104 (26.1)<br>170 (42.7)<br>25 (6.3)<br>25 (6.3)<br>46 (11.6)

Author, Year	Outcomes	Criteria		Age of participants (years old)		Sex of participants			Comorbidity of participants		
		Inclusion	Exclusion	Interventions	Comparators	Sex	Interventions	Comparators	Diseases	Interventions (%)	Comparators (%)
			years of age, and those with incomplete past medical history were also excluded.						Heart failure<br>Cancer<br>Immunosuppression<br>Chronic Kidney disease	22 (26.8)<br>7 (8.5)<br>8 (9.8)<br>14 (17.1)	10 (2.5)<br>25 (6.3)<br>22 (5.5)<br>28 (7.1)
Zheng Y. (2020)	Severe/non-severe	73 patients diagnosed with COVID -19 in Taihe hospital were identified as the research subjects. Epidemiological history, clinical manifestation, imaging	-	43		Male	40 (54.8%)		Cardiovascular disease<br>Endocrine disease<br>Respiratory disease<br>Digestive system disease<br>Malignant tumor<br>Other	12 (16.4)<br>4 (5.5)<br>4 (5.5)<br>3 (4.1)<br>1 (1.4)<br>8 (11.0)	

Author, Year	Outcomes	Criteria		Age of participants (years old)		Sex of participants			Comorbidity of participants		
		Inclusion	Exclusion	Interventions	Comparators	Sex	Interventions	Comparators	Diseases	Interventions (%)	Comparators (%)
		detection and Q-PCR of the respiratory specimens as tools to confirm positive cases.									
Zhou F. (2020)	Dead/alive	All adult inpatients ≥ 18 years old with laboratory confirmed COVID-19 were enrolled in the study.	-	69	52	Male	38 (70%)	81 (59%)	Hypertension<br>Diabetes mellitus<br>Coronary heart disease<br>Chronic obstructive pulmonary disease<br>Carcinoma<br>Chronic kidney disease<br>Other	26 (48)<br>17 (31)<br>13 (24)<br>4 (7)<br>0 (0.0)<br>2 (4)<br>11 (20)	32 (23)<br>19 (14)<br>2 (1)<br>2 (1)<br>2 (1)<br>0 (0.0)<br>11 (8)
Zinellu (2020)	Dead/alive	105 consecutive	-	79.5	68	Male	20 (71.4%)	27 (54%)	Cardiovascular disease	6	40

Author, Year	Outcomes	Criteria		Age of participants (years old)		Sex of participants			Comorbidity of participants		
		Inclusion	Exclusion	Interventions	Comparators	Sex	Interventions	Comparators	Diseases	Interventions (%)	Comparators (%)
		COVID-19 patients admitted to the Respiratory Disease and Infectious Disease Units of the University Hospital of Sassari and the Pneumology Unit of the Santissima Trinità Hospital of Cagliari, Sardinia, Italy, between 15 March and							Respiratory disease<br>Kidney disease<br>Diabetes<br>Cancer<br>Autoimmunity	20<br>25<br>21<br>24<br>27	62<br>65<br>62<br>65<br>72

Author, Year	Outcomes	Criteria		Age of participants (years old)		Sex of participants			Comorbidity of participants		
		Inclusion	Exclusion	Interventions	Comparators	Sex	Interventions	Comparators	Diseases	Interventions (%)	Comparators (%)
		15 May 2020. COVID-19 was confirmed by reverse transcription-polymerase chain reaction (RT-PCR) in all cases									

Table S5. Meta-regression analysis of the heterogeneous findings

Moderator	Outcome: Severe			Outcome: Dead		
	OR	95% CI	p-value	OR	95% CI	p-value
Hypertension	1.31	0.48-3.55	0.579	1.22	0.67-2.20	0.492
Diabetes Mellitus	0.55	0.20-1.53	0.232	0.99	0.62-1.60	0.973
Age over 65 years [a]	NA	NA	NA	1.00	0.57-1.74	0.998

OR, Odds ratio; 95% CI, 95% confidence interval; NA, not available
[a] reference was age less than 65 years

Table S6. Excluded studies

Reference	Reason for exclusion	Reference	Reason for exclusion
Abate B.B.[1]	Other study design	Münzel T.[2]	Other study design
Abohamr S.I.[3]	Other study design	Nasrolahi A.[4]	Other study design
Abohamr S.I.[5]	Duplicates participants	Naziroğlu T.[6]	Other study design
Abrams E.M.[7]	Other study design	Onisoyonivosekume D.[8]	Other study design
Acheampong D.O.[9]	Other study design	Pan J.[10]	Inappropriate outcomes
Adrish M.[11]	Inappropriate outcomes	Patanavanich R.[12]	Other study design
Aksu K.[13]	Inappropriate comparison	Patel U.[14]	Other study design
Alguwaihes A.M.[15]	Inappropriate comparison	Patterson T.J.[16]	Other study design
Almalki Z.S.[17]	Inappropriate outcomes	Pettigrew S.[18]	Inappropriate outcomes
Alomari S.O.[19]	Inappropriate outcomes	Polverino F.[20]	Inappropriate outcomes
Andreu-Mondon M.[21]	Other study design	Popova L.[22]	Other study design
Balaban Kocas B.[23]	Inappropriate outcomes	Purkayastha A.[24]	Inappropriate outcomes
Baldock T.E.[25]	Other study design	Radwan N.M.[26]	Other study design
Barlow P.[27]	Other study design	Risso D.[28]	Inappropriate outcomes
Best J.H.[29]	Inappropriate comparison	Rodriguez J.A.[30]	Other study design
Brendish N.J.[31]	Inappropriate outcomes	Rodriguez-Leor O.[32]	Inappropriate comparison
Castillo-López I.Y.[33]	Other study design	Salah H.M.[34]	Other study design
Chene G.[35]	Other study design	Scholz J.R.[36]	Other study design
Colaneri M.[37]	Inappropriate comparison	Schultze A.[38]	Inappropriate comparison
Costantino F.[39]	Inappropriate outcomes	Seiler N.K.[40]	Inappropriate comparison
Crisan-Dabija R.[41]	Other study design	Shahzad Hasan S.[42]	Other study design
Di Lecce V.[43]	Inappropriate outcomes	Sharma P.[44]	Inappropriate outcomes
Di Vincenzo A.[45]	Other study design	Shastri M.D.[46]	Other study design
Dorjee K.[47]	Other study design	Shatla M.M.[48]	Inappropriate outcomes
Dratcu L.[49]	Other study design	Shoar S.[50]	Other study design
Feyaerts A.F.[51]	Other study design	Shukla S.D.[52]	Inappropriate comparison
Fillmore N.R.[53]	Inappropriate comparison	Siddiqi K.[54]	Other study design
Fisman D.N.[55]	Inappropriate comparison	Sifat A.E.[56]	Other study design
Gaiha S.M.[57]	Inappropriate outcomes	Simons D.[58]	Other study design

Reference	Reason for exclusion	Reference	Reason for exclusion
Gallo O.[59]	Other study design	Sims O.T.[60]	Inappropriate outcomes
Gandhi S.A.[61]	Other study design	Sinclair R.G.[62]	Inappropriate comparison
Ghinai I.[63]	Inappropriate outcomes	Singh S.[64]	Other study design
González-Rubio J.[65]	Other study design	Soule E.K.[66]	Inappropriate outcomes
Grover S.[67]	Other study design	Streck J.M.[68]	Inappropriate comparison
Gu T.[69]	Duplicates participants	Takagi H.[70]	Other study design
Gupta A.K.[71]	Other study design	Talavera B.[72]	Inappropriate outcomes
Hashmi S.K.[73]	Other study design	Toussie D.[74]	Inappropriate comparison
He S.[75]	Inappropriate comparison	Tran T.V.[76]	Inappropriate comparison
Holman N.[77]	Inappropriate comparison	Tzu-Hsuan Chen D.[78]	Inappropriate outcomes
Horii T.[79]	Other study design	Underner M.[80]	Other study design
Hu J.[81]	Inappropriate outcomes	Urigo C.[82]	Other study design
Ieng S.M.[83]	Inappropriate comparison	Uvais N.A.[84]	Other study design
Ioannidis J.[85]	Other study design	Veldtman G.R.[86]	Inappropriate outcomes
Islam M.Z.[87]	Inappropriate comparison	Vila-Córcoles A.[88]	Inappropriate comparison
Izquierdo J.L.[89]	Inappropriate comparison	Volino-Souza M.[90]	Inappropriate comparison
Jourdes A.[91]	Inappropriate comparison	Vuolo M.[92]	Inappropriate outcomes
Landoni G.[93]	Other study design	Wang Z.[94]	Inappropriate comparison
Lang A.E.[95]	Other study design	Wenzl T.[96]	Other study design
Lang A.E.[97]	Other study design	Xiang J.[98]	Inappropriate comparison
Li J.[99]	Other study design	Xiong G.L.[100]	Inappropriate outcomes
Liu J.[101]	Inappropriate outcomes	Yue L.[102]	Other study design
Lu J.[103]	Inappropriate outcomes	Zajacova A.[104]	Other study design
Marvisi M.[105]	Inappropriate comparison	Zayet S.[106]	Other study design
McRobbie H.[107]	Other study design	Zeng L.[108]	Other study design
Meini S.[109]	Inappropriate comparison	Zhao Q.[110]	Other study design
Mesas A.E.[111]	Other study design	Zhou W.[112]	Inappropriate outcomes

Reference (excluded studies)

1. Abate BB, Kassie AM, Kassaw MW, Aragie TG, Masresha SA. Sex difference in coronavirus disease (COVID-19): a systematic review and meta-analysis. *BMJ Open*. 2020;10(10):e040129. doi:10.1136/bmjopen-2020-040129

2. Münzel T, Hahad O, Kuntic M, Keaney JF, Deanfield JE, Daiber A. Effects of tobacco cigarettes, e-cigarettes, and waterpipe smoking on endothelial function and clinical outcomes. *Eur Heart J*. 2020;41(41):4057-4070. doi:10.1093/eurheartj/ehaa460

3. Abohamr SI, Abazid RM, Aldossari MA, et al. Clinical characteristics and in-hospital mortality of COVID-19 adult patients in Saudi Arabia. *Saudi Med J*. 2020;41(11):1217-1226. doi:10.15537/smj.2020.11.25495

4. Nasrolahi A, Haghani K, Gheysarzadeh A, Bakhtiyari S. Do genetic factors predispose people to COVID-19: A review article. *Curr Mol Med*. Published online November 2020. doi:10.2174/1566524020999201113102145

5. Abohamr SI, Aldossari MA, Alaklobi FA, et al. Clinical characteristics and in-hospital outcome of medical staff infected with COVID-19 in Saudi Arabia. A retrospective single-center study. *Saudi Med J*. 2020;41(12):1336-1343. doi:10.15537/smj.2020.12.25514

6. Naziroğlu T, Aksu K. Rare atopy in COVID-19 patients or COVID-19 famine in atopic patients? *Dermatol Ther*. Published online November 2020. doi:10.1111/dth.14581

7. Abrams EM, Sinha I, Fernandes RM, Hawcutt DB. Pediatric asthma and COVID-19: The known, the unknown, and the controversial. *Pediatr Pulmonol*. 2020;55(12):3573-3578. doi:10.1002/ppul.25117

8. Onisoyonivosekume D, Mahrouseh N, Varga O. Introduction to Health Law. *Stud Health Technol Inform*. 2020;274:10-18. doi:10.3233/SHTI200660

9. Acheampong DO, Barffour IK, Boye A, Aninagyei E, Ocansey S, Morna MT. Male predisposition to severe COVID-19: Review of evidence and potential therapeutic prospects. *Biomed Pharmacother*. 2020;131:110748. doi:10.1016/j.biopha.2020.110748

10. Pan J, St Pierre JM, Pickering TA, et al. Coronavirus Disease 2019 (COVID-19): A Modeling Study of Factors Driving Variation in Case Fatality Rate by Country. *Int J Environ Res Public Health*. 2020;17(21). doi:10.3390/ijerph17218189

11. Adrish M, Chilimuri S, Mantri N, et al. Association of smoking status with outcomes in hospitalised patients with COVID-19. *BMJ open Respir Res*. 2020;7(1). doi:10.1136/bmjresp-2020-000716

12. Patanavanich R, Glantz SA. The Theoretical Problems Do Not Materially Affect the Results of Our Meta-analysis of Smoking and Covid-19 Disease Progression. *Nicotine Tob Res Off J Soc Res Nicotine Tob*. Published online November 2020. doi:10.1093/ntr/ntaa250

13. Aksu K, Naziroğlu T, Özkan P. Factors determining COVID-19 pneumonia severity in a country with routine BCG vaccination. *Clin Exp Immunol*. 2020;202(2):220-225.

doi:10.1111/cei.13507

14. Patel U, Malik P, Mehta D, et al. Early epidemiological indicators, outcomes, and interventions of COVID-19 pandemic: A systematic review. *J Glob Health*. 2020;10(2):20506. doi:10.7189/jogh.10.020506

15. Alguwaihes AM, Al-Sofiani ME, Megdad M, et al. Diabetes and Covid-19 among hospitalized patients in Saudi Arabia: a single-centre retrospective study. *Cardiovasc Diabetol*. 2020;19(1):205. doi:10.1186/s12933-020-01184-4

16. Patterson TJ, Currie PJ, Beck J, Spence RAJ, Spence GM. A systematic review of viral transmission risk to healthcare staff comparing laparoscopic and open surgery. *Surgeon*. 2020;18(6):e72-e77. doi:10.1016/j.surge.2020.06.016

17. Almalki ZS, Khan MF, Almazrou S, et al. Clinical Characteristics and Outcomes Among COVID-19 Hospitalized Patients with Chronic Conditions: A Retrospective Single-Center Study. *J Multidiscip Healthc*. 2020;13:1089-1097. doi:10.2147/JMDH.S273918

18. Pettigrew S, Jun M, Roberts I, Bullen C, Nallaiah K, Rodgers A. Preferences for Tobacco Cessation Information and Support During Covid-19. *J Addict Med*. 2020;14(6):e362-e365. doi:10.1097/ADM.0000000000000743

19. Alomari SO, Abou-Mrad Z, Bydon A. COVID-19 and the central nervous system. *Clin Neurol Neurosurg*. 2020;198:106116. doi:10.1016/j.clineuro.2020.106116

20. Polverino F, Stern DA, Ruocco G, et al. Comorbidities, Cardiovascular Therapies, and COVID-19 Mortality: A Nationwide, Italian Observational Study (ItaliCO). *Front Cardiovasc Med*. 2020;7:585866. doi:10.3389/fcvm.2020.585866

21. Andreu-Mondon M, Barrio-Gimenez P, Mondon-Vehils S. COVID-19 and restrictions on tobacco use. *Med Clin (Barc)*. Published online November 2020. doi:10.1016/j.medcli.2020.10.008

22. Popova L. Carpe covid: using COVID-19 to communicate about harms of tobacco products. *Tob Control*. Published online November 2020. doi:10.1136/tobaccocontrol-2020-056276

23. Balaban Kocas B, Cetinkal G, Ser OS, et al. The relation between left ventricular global longitudinal strain and troponin levels in patients hospitalized with COVID-19 pneumonia. *Int J Cardiovasc Imaging*. Published online November 2020:1-9. doi:10.1007/s10554-020-02102-1

24. Purkayastha A, Sen C, Garcia GJ, et al. Direct Exposure to SARS-CoV-2 and Cigarette Smoke Increases Infection Severity and Alters the Stem Cell-Derived Airway Repair Response. *Cell Stem Cell*. 2020;27(6):869-875.e4. doi:10.1016/j.stem.2020.11.010

25. Baldock TE, Bolam SM, Gao R, et al. Infection prevention measures for orthopaedic departments during the COVID-2019 pandemic: a review of current evidence. *Bone Jt open*. 2020;1(4):74-79. doi:10.1302/2633-1462.14.BJO-2020-0018.R1

26. Radwan NM, Mahmoud NE, Alfaifi AH, Alabdulkareem KI. Comorbidities and severity of coronavirus disease 2019 patients. *Saudi Med J*. 2020;41(11):1165-1174.

doi:10.15537/smj.2020.11.25454

27. Barlow P. COVID-19, Trade, and Health: This Changes Everything? Comment on "What Generates Attention to Health in Trade Policy-Making? Lessons From Success in Tobacco Control and Access to Medicines: A Qualitative Study of Australia and the (Comprehensive and Prog. *Int J Heal policy Manag*. Published online November 2020. doi:10.34172/ijhpm.2020.220

28. Risso D, Drayna D, Morini G. Alteration, Reduction and Taste Loss: Main Causes and Potential Implications on Dietary Habits. *Nutrients*. 2020;12(11). doi:10.3390/nu12113284

29. Best JH, Mohan S V, Kong AM, et al. Baseline Demographics and Clinical Characteristics Among 3471 US Patients Hospitalized with COVID-19 and Pulmonary Involvement: A Retrospective Study. *Adv Ther*. 2020;37(12):4981-4995. doi:10.1007/s12325-020-01510-y

30. Rodriguez JA, Roa AA, Lemos-Ramirez JC. E-Cigarette or Vaping Product Use-Associated Lung Injury (EVALI) Mimicking COVID-19 Disease. *Case Rep Pulmonol*. 2020;2020:8821289. doi:10.1155/2020/8821289

31. Brendish NJ, Poole S, Naidu V V, et al. Clinical characteristics, symptoms and outcomes of 1054 adults presenting to hospital with suspected COVID-19: A comparison of patients with and without SARS-CoV-2 infection. *J Infect*. 2020;81(6):937-943. doi:10.1016/j.jinf.2020.09.033

32. Rodriguez-Leor O, Cid Alvarez AB, de Prado AP, et al. In-hospital outcomes of patients with ST-segment elevation myocardial infarction and COVID-19. *EuroIntervention J Eur Collab with Work Gr Interv Cardiol Eur Soc Cardiol*. Published online November 2020. doi:10.4244/EIJ-D-20-00935

33. Castillo-López IY, Govea-Camacho LH, Rodríguez-Torres IA, Recio-Macías DA, Alobid I, Mullol J. Olfactory Dysfunction in a Mexican Population Outside of COVID-19 Pandemic: Prevalence and Associated Factors (the OLFAMEX Study). *Curr Allergy Asthma Rep*. 2020;20(12):78. doi:10.1007/s11882-020-00975-9

34. Salah HM, Sharma T, Mehta J. Smoking Doubles the Mortality Risk in COVID-19: A Meta-Analysis of Recent Reports and Potential Mechanisms. *Cureus*. 2020;12(10):e10837. doi:10.7759/cureus.10837

35. Chene G, Bouvet L, Cerruto E, Nohuz E. How can we minimize the potential risk of viral contamination during laparoscopic procedures for suspected or infected COVID-19 patients? *Eur J Obstet Gynecol Reprod Biol*. 2020;255:63-66. doi:10.1016/j.ejogrb.2020.10.011

36. Scholz JR, Lopes MACQ, Saraiva JFK, Colombo FC. COVID-19, Renin-Angiotensin System, Angiotensin-Converting Enzyme 2, and Nicotine: What is the Interrelation? *Arq Bras Cardiol*. 2020;115(4):708-711. doi:10.36660/abc.20200653

37. Colaneri M, Novelli V, Cutti S, et al. The experience of the health care workers of a severely hit SARS-CoV-2 referral Hospital in Italy: incidence, clinical course and modifiable risk factors for COVID-19 infection. *J Public Health (Oxf)*. Published online November 2020. doi:10.1093/pubmed/fdaa195

38. Schultze A, Walker AJ, MacKenna B, et al. Risk of COVID-19-related death among patients with chronic obstructive pulmonary disease or asthma prescribed inhaled corticosteroids: an observational cohort study using the OpenSAFELY platform. *Lancet Respir Med*. 2020;8(11):1106-1120. doi:10.1016/S2213-2600(20)30415-X

39. Costantino F, Bahier L, Tarancón LC, et al. COVID-19 in French patients with chronic inflammatory rheumatic diseases: clinical features, risk factors and treatment adherence. *Jt bone spine*. Published online November 2020:105095. doi:10.1016/j.jbspin.2020.105095

40. Seiler NK, Swamy R, Xiao J, Yun Y. Tobacco smoking cessation in mental health services during the COVID-19 pandemic. *J Addict Dis*. 2020;38(4):582-584. doi:10.1080/10550887.2020.1791379

41. Crisan-Dabija R, Pavel CA, Popa IV, Tarus A, Burlacu A. "A Chain Only as Strong as Its Weakest Link": An Up-to-Date Literature Review on the Bidirectional Interaction of Pulmonary Fibrosis and COVID-19. *J Proteome Res*. 2020;19(11):4327-4338. doi:10.1021/acs.jproteome.0c00387

42. Shahzad Hasan S, Kow CS, Ahmadi K. Smoking Cessation Amid the Coronavirus 2019 Pandemic: Making Every Contact Count. *Chronic Obstr Pulm Dis (Miami, Fla)*. 2020;7(4):300-302. doi:10.15326/jcopdf.7.4.2020.0171

43. Di Lecce V, Carpagnano GE, Pierucci P, et al. Baseline characteristics and outcomes of COVID-19 patients admitted to a Respiratory Intensive Care Unit (RICU) in Southern Italy. *Multidiscip Respir Med*. 2020;15(1):704. doi:10.4081/mrm.2020.704

44. Sharma P, Ebbert JO, Rosedahl JK, Philpot LM. Changes in substance use among young adults during a respiratory disease pandemic. *SAGE open Med*. 2020;8:2050312120965321. doi:10.1177/2050312120965321

45. Di Vincenzo A, Vettor R, Rossato M. COVID-19 and smoking habits: a smoky situation! *Monaldi Arch chest Dis = Arch Monaldi per le Mal del torace*. 2020;90(4). doi:10.4081/monaldi.2020.1539

46. Shastri MD, Shukla SD, Chong WC, et al. Smoking and COVID-19: What we know so far. *Respir Med*. 2020;176:106237. doi:10.1016/j.rmed.2020.106237

47. Dorjee K, Kim H, Bonomo E, Dolma R. Prevalence and predictors of death and severe disease in patients hospitalized due to COVID-19: A comprehensive systematic review and meta-analysis of 77 studies and 38,000 patients. *PLoS One*. 2020;15(12):e0243191. doi:10.1371/journal.pone.0243191

48. Shatla MM, Khafagy AA, Bulkhi AA, Aljahdali IA. Public Concerns and Mental Health Changes Related to the COVID-19 Pandemic Lockdown in Saudi Arabia. *Clin Lab*. 2020;66(10). doi:10.7754/Clin.Lab.2020.200614

49. Dratcu L, Boland X. Does Nicotine Prevent Cytokine Storms in COVID-19? *Cureus*. 2020;12(10):e11220. doi:10.7759/cureus.11220

50. Shoar S, Hosseini F, Naderan M, Mehta JL. Meta-analysis of Cardiovascular Events and Related Biomarkers Comparing Survivors Versus Non-survivors in Patients With COVID-19. *Am J Cardiol*. 2020;135:50-61. doi:10.1016/j.amjcard.2020.08.044

51. Feyaerts AF, Luyten W. Vitamin C as prophylaxis and adjunctive medical treatment for COVID-19? *Nutrition*. 2020;79-80:110948. doi:10.1016/j.nut.2020.110948

52. Shukla SD, O'Toole RF. Exposure to bushfire and biomass smoke and the risk of bacterial and viral lung infection. *Respirology*. 2020;25(11):1121-1122. doi:10.1111/resp.13908

53. Fillmore NR, La J, Szalat RE, et al. Prevalence and outcome of COVID-19 infection in cancer patients: a national Veterans Affairs study. *J Natl Cancer Inst*. Published online October 2020. doi:10.1093/jnci/djaa159

54. Siddiqi K, Siddiqui F, Khan A, et al. The impact of COVID-19 on smoking patterns in Pakistan: findings from a longitudinal survey of smokers. *Nicotine Tob Res Off J Soc Res Nicotine Tob*. Published online October 2020. doi:10.1093/ntr/ntaa207

55. Fisman DN, Greer AL, Hillmer M, Tuite R. Derivation and Validation of Clinical Prediction Rules for COVID-19 Mortality in Ontario, Canada. *Open forum Infect Dis*. 2020;7(11):ofaa463. doi:10.1093/ofid/ofaa463

56. Sifat AE, Nozohouri S, Villalba H, Vaidya B, Abbruscato TJ. The Role of Smoking and Nicotine in the Transmission and Pathogenesis of COVID-19. *J Pharmacol Exp Ther*. 2020;375(3):498-509. doi:10.1124/jpet.120.000170

57. Gaiha SM, Cheng J, Halpern-Felsher B. Association Between Youth Smoking, Electronic Cigarette Use, and COVID-19. *J Adolesc Heal Off Publ Soc Adolesc Med*. 2020;67(4):519-523. doi:10.1016/j.jadohealth.2020.07.002

58. Simons D, Shahab L, Brown J, Perski O. The association of smoking status with SARS-CoV-2 infection, hospitalization and mortality from COVID-19: a living rapid evidence review with Bayesian meta-analyses (version 7). *Addiction*. Published online October 2020. doi:10.1111/add.15276

59. Gallo O. Risk for COVID-19 infection in patients with tobacco smoke-associated cancers of the upper and lower airway. *Eur Arch oto-rhino-laryngology Off J Eur Fed Oto-Rhino-Laryngological Soc Affil with Ger Soc Oto-Rhino-Laryngology - Head Neck Surg*. Published online November 2020:1-8. doi:10.1007/s00405-020-06456-z

60. Sims OT, Jackson A, Guo Y, Truong DN, Odame EA, Mamudu HM. A Cross-Sectional Analysis of Tobacco Use and Concurrent Alcohol and Substance Use Among Patients Living with HIV/HCV Co-infection: Findings from a Large Urban Tertiary Center. *J Clin Psychol Med Settings*. Published online October 2020:1-9. doi:10.1007/s10880-020-09744-2

61. Gandhi SA. Letter from California, USA. *Respirology*. Published online November 2020. doi:10.1111/resp.13980

62. Sinclair RG, Somsamouth K, Sahar D, Englert R, Singh P. Microbial contamination in the communal-use Lao tobacco waterpipe. *Int Health*. Published online October 2020. doi:10.1093/inthealth/ihaa078

63. Ghinai I, Davis ES, Mayer S, et al. Risk Factors for Severe Acute Respiratory Syndrome Coronavirus 2 Infection in Homeless Shelters in Chicago, Illinois-March-May, 2020. *Open forum Infect Dis*. 2020;7(11):ofaa477. doi:10.1093/ofid/ofaa477

64. Singh S, Zuwasti U, Haas C. Coronavirus-Associated Coagulopathy: Lessons From SARS-CoV1 and MERS-CoV for the Current SARS-CoV2 Pandemic. *Cureus*. 2020;12(11):e11310. doi:10.7759/cureus.11310

65. González-Rubio J, Navarro-López C, López-Nájera E, et al. A Systematic Review and Meta-Analysis of Hospitalised Current Smokers and COVID-19. *Int J Environ Res Public Health*. 2020;17(20). doi:10.3390/ijerph17207394

66. Soule EK, Kheradmand F, Eissenberg T. Health practitioners should caution about misinformation and association of adverse effects of electronic cigarette use and COVID-19. *Prev Med reports*. 2020;20:101255. doi:10.1016/j.pmedr.2020.101255

67. Grover S, Mohanty V, Jain S, Anand T, Aghi MB. "YES it's the Perfect Time to Quit": Fueling Tobacco Cessation in India during COVID-19 Pandemic. *Tob use insights*. 2020;13:1179173X20960447. doi:10.1177/1179173X20960447

68. Streck JM, Kalkhoran S, Bearnot B, et al. Perceived risk, attitudes, and behavior of cigarette smokers and nicotine vapers receiving buprenorphine treatment for opioid use disorder during the COVID-19 pandemic. *Drug Alcohol Depend*. 2020;218:108438. doi:10.1016/j.drugalcdep.2020.108438

69. Gu T, Mack JA, Salvatore M, et al. Characteristics Associated With Racial/Ethnic Disparities in COVID-19 Outcomes in an Academic Health Care System. *JAMA Netw open*. 2020;3(10):e2025197. doi:10.1001/jamanetworkopen.2020.25197

70. Takagi H. Systematic review of the prevalence of current smoking among hospitalized COVID-19 patients in China: could nicotine be a therapeutic option? *Intern Emerg Med*. 2020;15(8):1601-1603. doi:10.1007/s11739-020-02473-2

71. Gupta AK, Nethan ST, Mehrotra R. Tobacco use as a well-recognized cause of severe COVID-19 manifestations. *Respir Med*. 2020;176:106233. doi:10.1016/j.rmed.2020.106233

72. Talavera B, García-Azorín D, Martínez-Pías E, et al. Anosmia is associated with lower in-hospital mortality in COVID-19. *J Neurol Sci*. 2020;419:117163. doi:10.1016/j.jns.2020.117163

73. Hashmi SK, Hussain F, Hays JT. Thank You for Not Smoking. *Mayo Clin Proc*. 2020;95(10):2062-2064. doi:10.1016/j.mayocp.2020.08.012

74. Toussie D, Voutsinas N, Finkelstein M, et al. Clinical and Chest Radiography Features Determine Patient Outcomes in Young and Middle-aged Adults with COVID-19. *Radiology*. 2020;297(1):E197-E206. doi:10.1148/radiol.2020201754

75. He S, Tian J, Li X, et al. Positive RT-PCR Test Results in 420 Patients Recovered From COVID-19 in Wuhan: An Observational Study. *Front Pharmacol*. 2020;11:549117. doi:10.3389/fphar.2020.549117

76. Tran T V, Nguyen HC, Pham L V, et al. Impacts and interactions of COVID-19 response involvement, health-related behaviours, health literacy on anxiety, depression and health-related quality of life among healthcare workers: a cross-sectional study. *BMJ Open*. 2020;10(12):e041394. doi:10.1136/bmjopen-2020-041394

77. Holman N, Knighton P, Kar P, et al. Risk factors for COVID-19-related mortality in

people with type 1 and type 2 diabetes in England: a population-based cohort study. *lancet Diabetes Endocrinol*. 2020;8(10):823-833. doi:10.1016/S2213-8587(20)30271-0

78. Tzu-Hsuan Chen D. The psychosocial impact of the COVID-19 pandemic on changes in smoking behavior: Evidence from a nationwide survey in the UK. *Tob Prev Cessat*. 2020;6:59. doi:10.18332/tpc/126976

79. Horii T, Fujioka T, Takahashi M, et al. Late-onset pneumothorax in a COVID-19 patient treated with ventilation and ECMO: A case report and literature review. *Radiol case reports*. 2020;15(12):2560-2564. doi:10.1016/j.radcr.2020.09.036

80. Underner M, Perriot J, Peiffer G, Jaafari N. [COVID-19 and changes in smoking behavior]. *Rev Mal Respir*. 2020;37(8):684-686. doi:10.1016/j.rmr.2020.08.004

81. Hu J, Zhou J, Dong F, et al. Combination of serum lactate dehydrogenase and sex is predictive of severe disease in patients with COVID-19. *Medicine (Baltimore)*. 2020;99(42):e22774. doi:10.1097/MD.0000000000022774

82. Urigo C, Soin S, Sahu A. Spontaneous pneumomediastinum as a complication of a COVID-19 related pneumonia: case report and review of literature. *Radiol case reports*. 2020;15(12):2577-2581. doi:10.1016/j.radcr.2020.09.052

83. Ieng SM, Cheong IH. An Overview of Epidemiology of COVID-19 in Macau S.A.R. *Front public Heal*. 2020;8:550057. doi:10.3389/fpubh.2020.550057

84. Uvais NA. Interests in quitting smoking and alcohol during COVID-19 pandemic in India: A Google Trends study. *Psychiatry Clin Neurosci*. 2020;74(10):550-551. doi:10.1111/pcn.13118

85. Ioannidis JPA, Jha P. Does the COVID-19 pandemic provide an opportunity to eliminate the tobacco industry? *Lancet Glob Heal*. Published online October 2020. doi:10.1016/S2214-109X(20)30466-6

86. Veldtman GR, Pirisi M, Storti E, et al. Management principles in patients with COVID-19: perspectives from a growing global experience with emphasis on cardiovascular surveillance. *Open Hear*. 2020;7(2). doi:10.1136/openhrt-2020-001357

87. Islam MZ, Riaz BK, Islam ANMS, et al. Risk factors associated with morbidity and mortality outcomes of COVID-19 patients on the 28th day of the disease course: a retrospective cohort study in Bangladesh. *Epidemiol Infect*. 2020;148:e263. doi:10.1017/S0950268820002630

88. Vila-Córcoles A, Ochoa-Gondar O, Satué-Gracia EM, et al. Influence of prior comorbidities and chronic medications use on the risk of COVID-19 in adults: a population-based cohort study in Tarragona, Spain. *BMJ Open*. 2020;10(12):e041577. doi:10.1136/bmjopen-2020-041577

89. Izquierdo JL, Almonacid C, González Y, et al. The Impact of COVID-19 on Patients with Asthma. *Eur Respir J*. Published online November 2020. doi:10.1183/13993003.03142-2020

90. Volino-Souza M, de Oliveira GV, Conte-Junior CA, Alvares TS. Covid-19 Quarantine: Impact of Lifestyle Behaviors Changes on Endothelial Function and

Possible Protective Effect of Beetroot Juice. *Front Nutr*. 2020;7:582210. doi:10.3389/fnut.2020.582210

91. Jourdes A, Lafaurie M, Martin-Blondel G, et al. Clinical characteristics and outcome of hospitalized patients with SARS-CoV-2 infection at Toulouse University hospital (France). Results from the Covid-clinic-Toul cohort. *La Rev Med interne*. 2020;41(11):732-740. doi:10.1016/j.revmed.2020.08.006

92. Vuolo M, Kelly BC, Roscigno VJ. COVID-19 Mask Requirements as a Workers' Rights Issue: Parallels to Smoking Bans. *Am J Prev Med*. 2020;59(5):764-767. doi:10.1016/j.amepre.2020.07.001

93. Landoni G, Maimeri N, Fedrizzi M, et al. Why are Asian countries outperforming the Western world in controlling COVID-19 pandemic? *Pathog Glob Health*. Published online November 2020:1-3. doi:10.1080/20477724.2020.1850982

94. Wang Z, Zheutlin A, Kao Y-H, et al. Hospitalised COVID-19 patients of the Mount Sinai Health System: a retrospective observational study using the electronic medical records. *BMJ Open*. 2020;10(10):e040441. doi:10.1136/bmjopen-2020-040441

95. Lang AE, Yakhkind A. Coronavirus Disease 2019 and Smoking: How and Why We Implemented a Tobacco Treatment Campaign. *Chest*. 2020;158(4):1770-1776. doi:10.1016/j.chest.2020.06.013

96. Wenzl T. Smoking and COVID-19: Did we overlook representativeness? *Tob Induc Dis*. 2020;18:89. doi:10.18332/tid/129584

97. Lang AE, Yakhkind A. More Than Meets the Eye: The Similarities Between Coronavirus Disease 2019 and Smoking. *Mayo Clin Proc*. 2020;95(10):2282-2283. doi:10.1016/j.mayocp.2020.08.008

98. Xiang J, Chen Z, Zhou J, et al. Comparative analysis of the main haematological indexes and RNA detection for the diagnosis of SARS-CoV-2 infection. *BMC Infect Dis*. 2020;20(1):779. doi:10.1186/s12879-020-05489-3

99. Li J, Long X, Zhang Q, et al. Tobacco smoking confers risk for severe COVID-19 unexplainable by pulmonary imaging. *J Intern Med*. Published online December 2020. doi:10.1111/joim.13190

100. Xiong GL, Atkin A, Moquin K, et al. COVID-19 Transmission in a Psychiatric Long-Term Care Rehabilitation Facility: An Observational Study. *Prim care companion CNS Disord*. 2020;22(6). doi:10.4088/PCC.20m02765

101. Liu J, Chen T, Yang H, et al. Clinical and radiological changes of hospitalised patients with COVID-19 pneumonia from disease onset to acute exacerbation: a multicentre paired cohort study. *Eur Radiol*. 2020;30(10):5702-5708. doi:10.1007/s00330-020-06916-4

102. Yue L, Zhang R, Duan G. The relationship between smoking and COVID-19 progression. *Nicotine Tob Res Off J Soc Res Nicotine Tob*. Published online November 2020. doi:10.1093/ntr/ntaa245

103. Lu J, Yin Q, Li Q, et al. Clinical characteristics and factors affecting the duration of positive nucleic acid test for patients of COVID-19 in XinYu, China. *J Clin Lab Anal*.

2020;34(10):e23534. doi:10.1002/jcla.23534

104. Zajacova A, Jehn A, Stackhouse M, Denice P, Ramos H. Changes in health behaviours during early COVID-19 and socio-demographic disparities: a cross-sectional analysis. *Can J Public Health*. 2020;111(6):953-962. doi:10.17269/s41997-020-00434-y

105. Marvisi M, Ferrozzi F, Balzarini L, Mancini C, Ramponi S, Uccelli M. First report on clinical and radiological features of COVID-19 pneumonitis in a Caucasian population: Factors predicting fibrotic evolution. *Int J Infect Dis IJID Off Publ Int Soc Infect Dis*. 2020;99:485-488. doi:10.1016/j.ijid.2020.08.054

106. Zayet S, Klopfenstein T, Mezher C, Gendrin V, Conrozier T, Ben Abdallah Y. Coronavirus disease 2019 with spontaneous pneumothorax, pneumomediastinum and subcutaneous emphysema, France. *New microbes new Infect*. 2020;38:100785. doi:10.1016/j.nmni.2020.100785

107. McRobbie H, Kwan B. Tobacco use disorder and the lungs. *Addiction*. Published online November 2020. doi:10.1111/add.15309

108. Zeng L, Wang S, Cai J, et al. Clinical characteristics of COVID-19 with cardiac injury: a systematic review and meta-analysis. *Epidemiol Infect*. 2020;148:e266. doi:10.1017/S0950268820002587

109. Meini S, Fortini A, Andreini R, Sechi LA, Tascini C. The Paradox of the Low Prevalence of Current Smokers Among Covid-19 Patients Hospitalized in Non-Intensive Care Wards: Results From an Italian Multicenter Case-Control Study. *Nicotine Tob Res Off J Soc Res Nicotine Tob*. Published online September 2020. doi:10.1093/ntr/ntaa188

110. Zhao Q, Meng M, Kumar R, et al. The impact of COPD and smoking history on the severity of COVID-19: A systemic review and meta-analysis. *J Med Virol*. 2020;92(10):1915-1921. doi:10.1002/jmv.25889

111. Mesas AE, Cavero-Redondo I, Álvarez-Bueno C, et al. Predictors of in-hospital COVID-19 mortality: A comprehensive systematic review and meta-analysis exploring differences by age, sex and health conditions. *PLoS One*. 2020;15(11):e0241742. doi:10.1371/journal.pone.0241742

112. Zhou W, Song L, Wang X, et al. Cardiac injury prediction and lymphocyte immunity and inflammation analysis in hospitalized patients with coronavirus disease 2019 (COVID-19). *Int J Cardiol*. Published online October 2020. doi:10.1016/j.ijcard.2020.10.049

Figures

Figure S1. Funnel plot of included studies (Severity; all age group, and ≤ 65 years old)

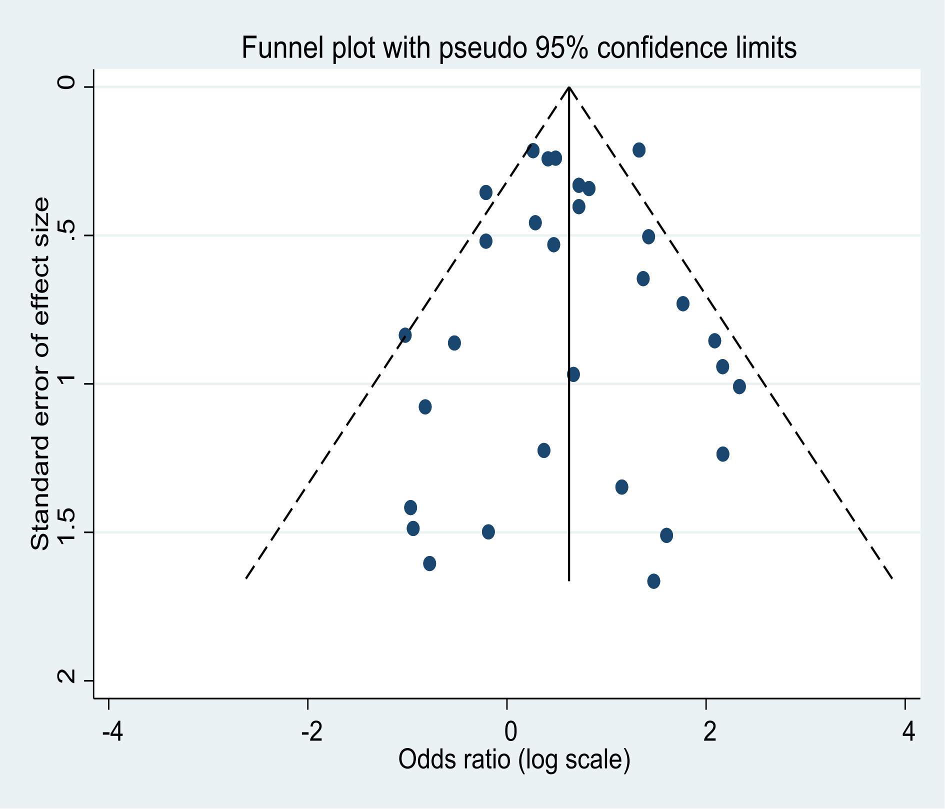

Figure S2. The graph from Egger's test of included studies in the meta-analysis (Severity; all age group, and ≤ 65 years old)

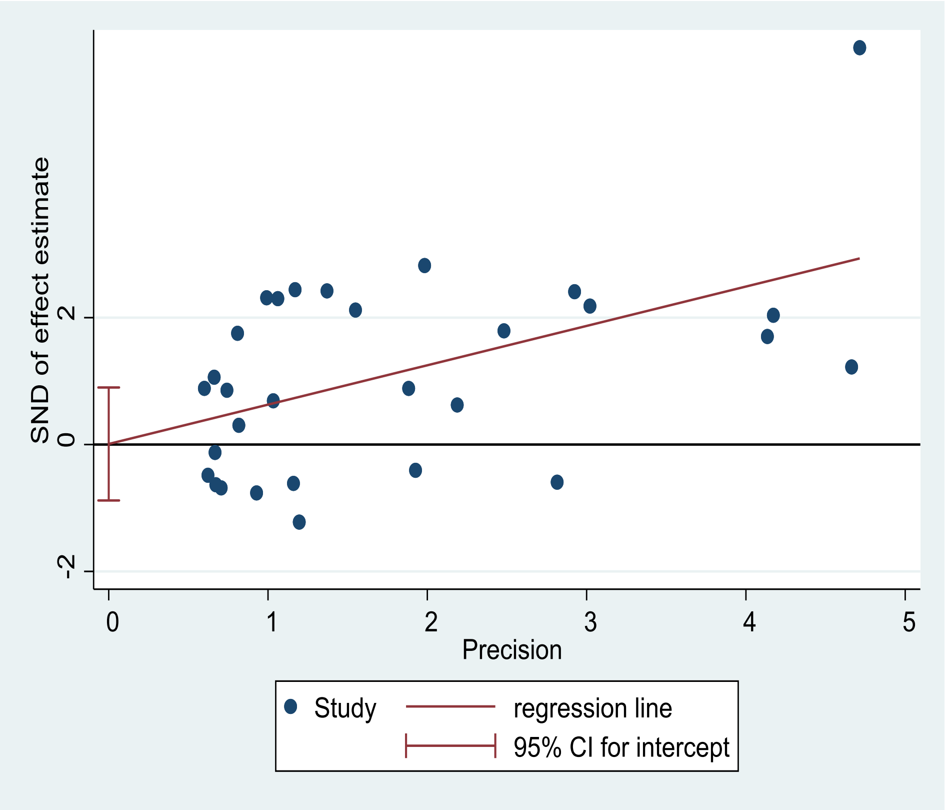

Figure S3. Funnel plot of included studies (Dead; all age group)

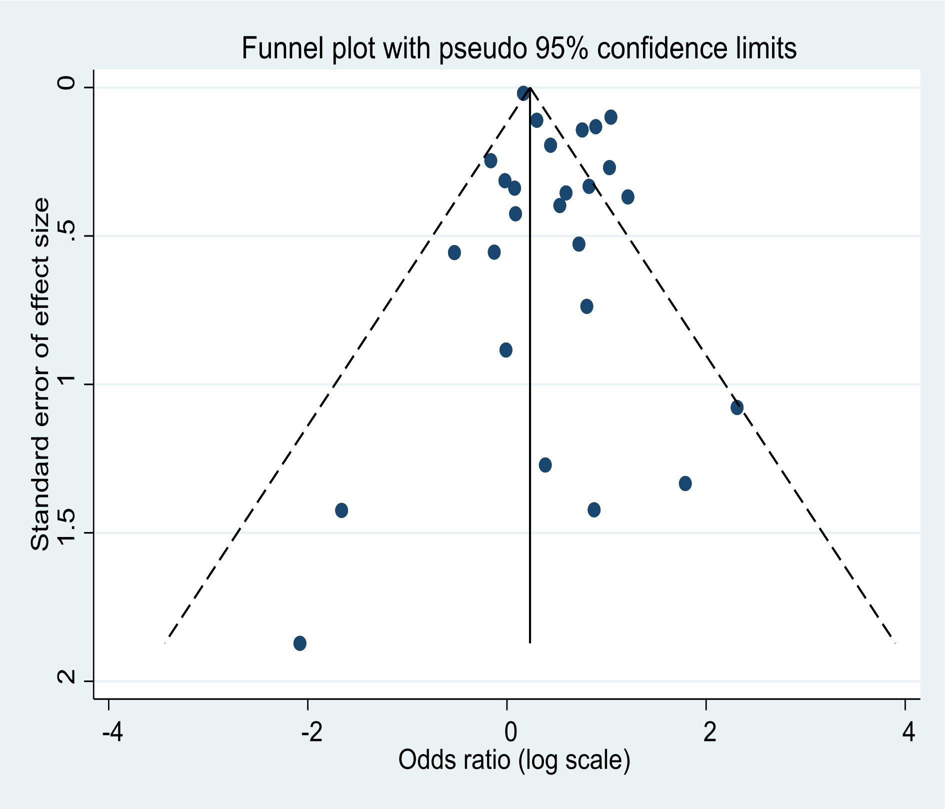

Figure S4. The graph from Egger's test of included studies in the meta-analysis (Dead; all age group)

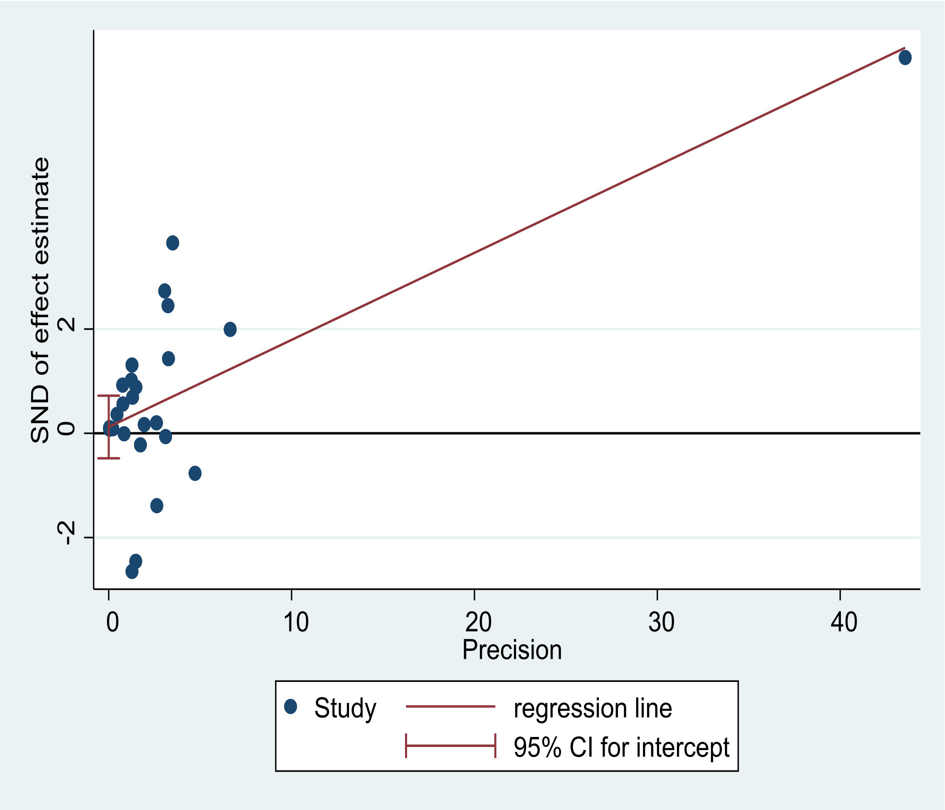

Figure S5. Funnel plot of included studies (Dead; ≤65 years old)

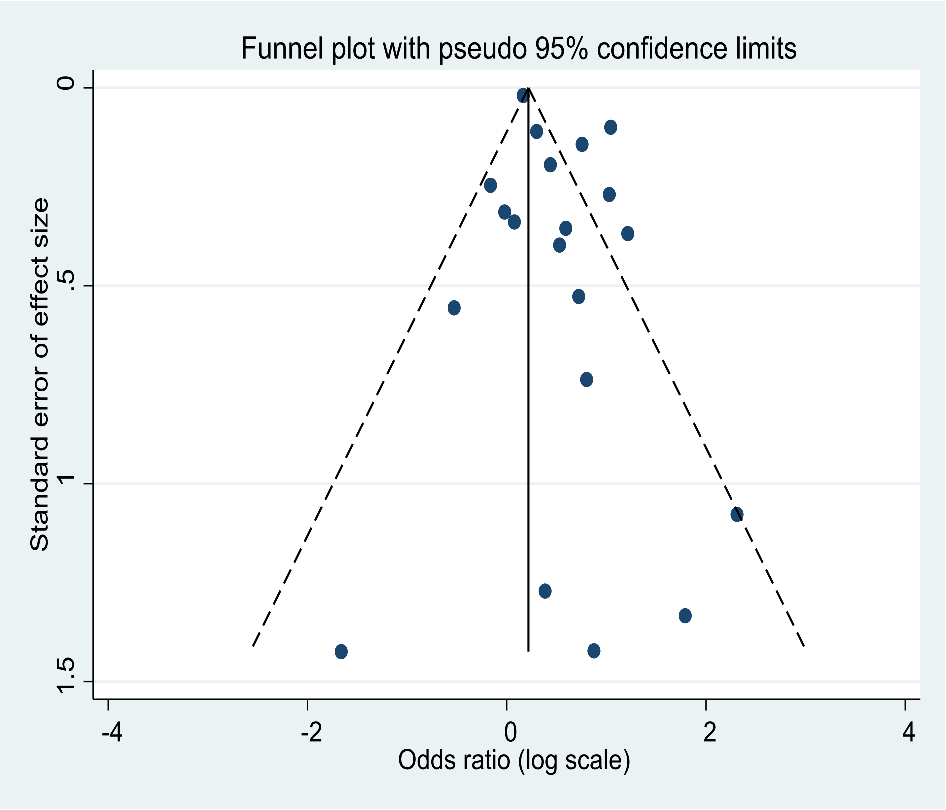

Figure S6. The graph from Egger's test of included studies in the meta-analysis (Dead; ≤65 years old)

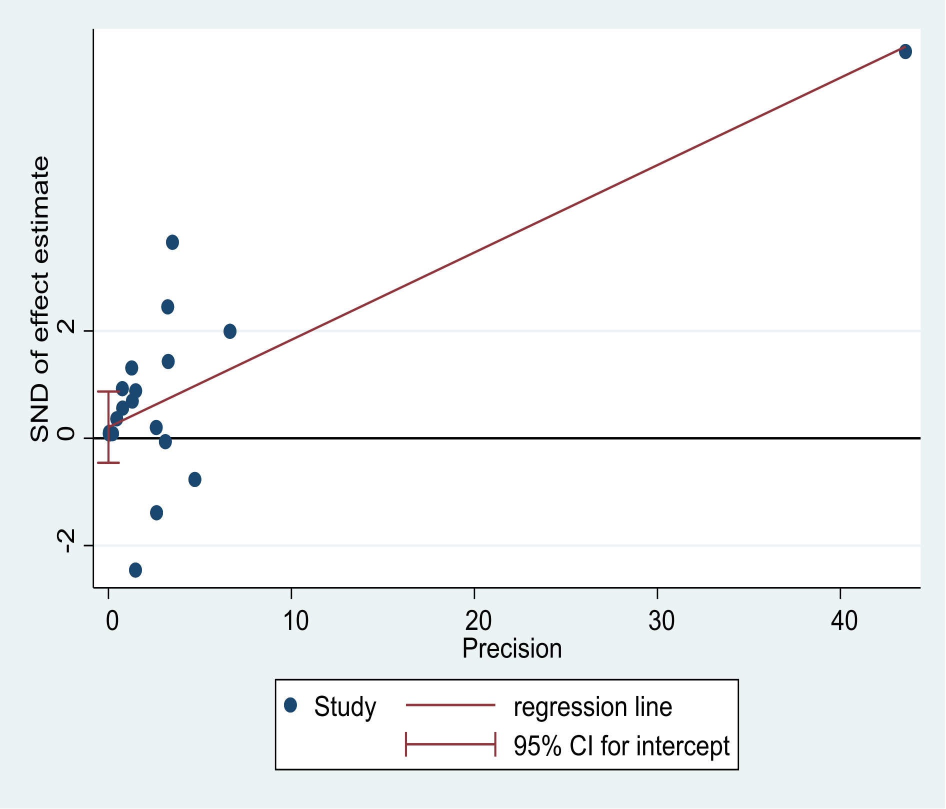

© 2021 Umnuaypornlert A. et al.
